# Simultaneous Cerebral Blood Flow and Cerebrovascular Reactivity Obtained From Novel Multi‐Band Multi‐Echo Pseudo‐Continuous Arterial Spin Labeling (M2‐PCASL) Sequence: A Test–Retest Reliability Study

**DOI:** 10.1002/mrm.70307

**Published:** 2026-02-18

**Authors:** Jody Todd, Maria‐Julieta Mateos, Ibraheem Budeir, Deqiang Qiu

**Affiliations:** ^1^ Department of Radiology and Imaging Sciences Emory University Atlanta Georgia USA; ^2^ Georgia Institute of Technology Atlanta Georgia USA

**Keywords:** arterial spin labeling, blood oxygen level‐dependent, cerebral blood flow, cerebrovascular reactivity, multi‐band, multi‐echo

## Abstract

**Purpose:**

We aim to assess the reliability of cerebral blood flow (CBF) and cerebrovascular reactivity (CVR) obtained simultaneously from a novel multi‐band pseudo‐continuous arterial spin labeling (M2‐PCASL) sequence and hypercapnic gas challenge, to examine factors influencing CBF variability, and to evaluate the impact of carbon dioxide (CO_2_) concentration on CVR estimates.

**Methods:**

Nine participants underwent test–retest M2‐PCASL scans at 3T with hypercapnia to quantify CBF and CVR. Reliability was assessed using the intraclass correlation coefficient (ICC) at whole brain, region of interest (ROI), and voxel levels. Linear mixed‐effect models were used to investigate factors contributing to CBF variability. Independent samples *t*‐tests were used to compare BOLD CVR and temporal contrast to noise ratio (tCNR) from different CO_2_ concentrations.

**Results:**

With smoothing, the M2‐PCASL sequence estimated baseline CBF and BOLD CVR with good to excellent reliability (ICC > 0.81) in the whole brain, gray matter (GM), and white matter (WM). Average ICCs across the Automated Anatomical Labeling atlas ROIs were between 0.50 ± 0.30 and 0.69 ± 0.19. CBF CVR achieved fair to moderate reliability in GM (ICCs between 0.42 and 0.55). Head motion was significantly associated with CBF temporal SNR (*p* ≤ 0.002). 5% and 8% CO_2_ yielded similar BOLD CVR estimates, but the BOLD tCNR using 5% CO_2_ was lower, although not statistically significant.

**Conclusion:**

The M2‐PCASL sequence with hypercapnia reliably estimates baseline CBF and BOLD CVR at improved spatial and temporal resolutions relative to existing methods, enabling us to noninvasively and comprehensively assess cerebrovascular health.

## 1 Introduction

Cerebrovascular integrity is altered in healthy aging and vascular and neurological diseases including Alzheimer's Disease (AD) and stroke [1, 2]. Arterial spin labeling (ASL) MRI has revealed impaired baseline cerebral blood flow (CBF) in cerebrovascular steno‐occlusive diseases [2, 3, 4]. While informative, baseline CBF alone is insufficient to holistically assess cerebrovascular function [5]. Studies have shown that cerebrovascular reactivity (CVR), a measure of the cerebrovasculature's capacity to respond to a vasodilatory stimulus, such as hypercapnia or acetazolamide, is also impaired in patients with cerebrovascular steno‐occlusive diseases [6, 7]. For example, subjects with recurrent cerebral ischemic events were found to exhibit significantly lower CVR than those with a single event, despite no differences in baseline CBF [1]. CVR was also found to predict cerebral ischemic events with an odds ratio of 14.4 (*p* = 0.0021) in asymptomatic patients with carotid occlusion and/or stenosis, independent of diabetes, hypertension, or smoking status [7]. Moreover, besides the hallmarks of amyloid and tau deposition in the brain, vascular dysfunction is increasingly recognized as a key contributor to AD pathophysiology. Epidemiological studies have shown that AD and cerebrovascular disease have a striking overlap in risk factors, including age, hypertension, diabetes, APOE4 status, hypercholesterolemia, and others [8, 9]. Increased cerebral atherosclerosis has been found in AD dementia patients compared to non‐demented controls [10, 11], and significant differences in CBF and arterial stiffness have been observed across stages of the AD continuum [12, 13, 14]. Cerebrovascular parameters could thus serve as valuable biomarkers for AD, complementing amyloid and tau, enhancing our understanding of its complex pathophysiology, and improving risk stratification [15]. Taken together, these findings indicate the need for robust and reliable methods for assessing multiple cerebrovascular parameters, including CBF and CVR, to comprehensively evaluate cerebrovascular health.

CBF and blood oxygen level‐dependent (BOLD) CVR measurements are complementary; however, most studies only assess either CBF or CVR. ASL‐derived CBF is quantitative and physiologically specific; although, it is temporally noisy because of the subtraction of control and tag acquisitions required for CBF quantification. In contrast, BOLD is less variable temporally and is sensitive to hemodynamic changes induced by vasodilatory stimuli. However, BOLD reflects the combined effects of CBF, cerebral blood volume, and oxygen metabolism [16] and is therefore less physiologically specific. The simultaneous acquisition of CBF and BOLD data can be performed using a dual‐echo ASL acquisition combined with a hypercapnic gas challenge. These techniques often have limited slice coverage due to constraints on the post‐label delay (PLD) and TR. For instance, Hoogeveen et al. were only able to acquire 15 slices with a slice thickness of 7 mm (TR = 4.1 s) [17].

This work aims to address limitations of traditional ASL/BOLD acquisitions by using a novel multi‐echo pseudo‐continuous ASL sequence with multi‐band (MB) acceleration (M2‐PCASL) combined with a hypercapnic gas challenge to simultaneously measure multiple cerebrovascular parameters. This builds upon our work presented as an abstract at ISMRM 2017 [18] where we demonstrated that MB acceleration in multi‐echo ASL enhances spatial and temporal resolutions, enabling the simultaneous acquisition of CBF and BOLD data with greater spatial coverage. Here, we evaluate the test–retest reliability of baseline CBF, BOLD CVR, and CBF CVR obtained from the M2‐PCASL sequence during an 8% CO_2_ gas challenge. We also compare the reliability of baseline CBF across different image resolutions and MB factors. Finally, we assess whether 8% CO_2_ offers sensitivity gains over the typical 5% CO_2_ stimulus.

## 2 Methods

### 2.1 Participants

Thirteen healthy subjects (age = 29.6 ± 7.2 years, 9 males) were recruited to undergo MR scans on a 3T Siemens Prisma scanner with the novel M2‐PCASL sequence during a hypercapnic gas challenge. All subjects provided written informed consent in accordance with a protocol approved by the Emory University Institutional Review Board. Each subject was scanned twice with a minimum and maximum duration between scans of 1 and 20 days, respectively. The time of day between scans was kept approximately consistent to control for physiological fluctuations that might occur throughout the day. Subjects refrained from caffeine 12 h prior to scans to control for its vasoconstrictive effects and improve reproducibility of hemodynamic measurements [19].

### 2.2 MRI Protocol and Hypercapnia Challenge

**FIGURE 1:**
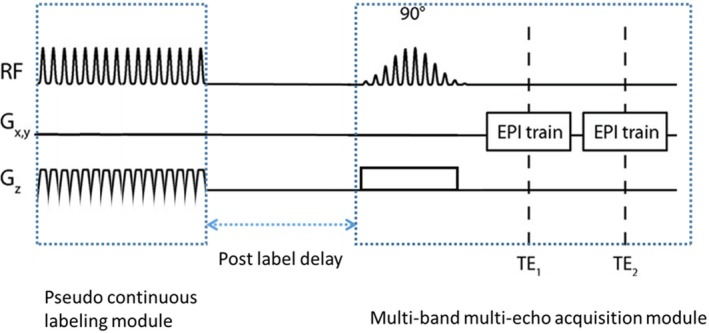
Multi‐band multi‐echo pseudo‐continuous arterial spin labeling (M2‐PCASL) sequence diagram with pseudo‐continuous labeling module and EPI readout.

In each session, subjects were scanned with the M2‐PCASL sequence (two echoes, TEs = 14/28 ms, TR = 4.064 s, flip angle = 90°, MB factor = 2, FOV = 224 mm^2^, matrix size = 64 × 64, resolution = 3.5 × 3.5 × 6 mm, 20 slices) (Figure 1) during a gas challenge that consisted of one 2‐min block of room air (21% oxygen, 79% nitrogen), one 2‐min block of 8% CO_2_, 21% oxygen and balanced nitrogen, and another 2‐min block of room air inhalation. End‐tidal CO_2_ (EtCO_2_) traces were monitored using a BIOPAC MP160 system with a CO2100C module (BIOPAC Systems Inc., Goleta, CA, USA). Subjects inhaled gases through a mouthpiece with a nose clip to prevent nasal inhalation. Following this scan, subjects were scanned with a high‐resolution variant of the M2‐PCASL sequence (three echoes, TEs = 8.8/20.6/32.3 ms, TR = 3.772 s, flip angle = 90°, MB factor = 3, FOV = 210 mm^2^, matrix size = 64 × 64, resolution = 3.3 × 3.3 × 3.3 mm, 39 slices) in the absence of a gas challenge. Baseline CBF was obtained from both M2‐PCASL acquisitions, while CVR derived from BOLD and CBF was obtained only from the lower resolution acquisition. The high‐resolution acquisition was included to compare the reliability of baseline CBF across different MB factors and image resolutions. To evaluate the effects of labeling duration (LD) on the tSNR of the CBF data, in both M2‐PCASL variants, we scanned 6 out of the 13 subjects with a LD of 1.5 s and the rest of the subjects with a LD of 1.8 s. We hypothesized that the spatial SNR gains from longer labeling [20] would translate to increased CBF tSNR. The PLD was 1.5 s for both acquisitions. A T_1_‐weighted MPRAGE sequence was included to visualize high‐resolution anatomical details (TE = 2.96 ms, TR = 2.3 s, flip angle = 90°, FOV = 224 mm^2^, matrix size = 208 × 240, resolution = 1 × 1 × 1 mm, 256 slices).

### 2.3 Multi‐Band Multi‐Echo Reconstruction

We enabled multi‐echo acquisition during the main imaging sequence as well as during the MB calibration scan, allowing for improved reconstruction and consistency across echoes. We formulate the MB multi‐echo reconstruction problem as follows:

(1) $$A_{z}K_{z}=B_{z}$$

where $A_{z}$ (dimension $N_{\text{echo}}N_{k}\times N_{\text{neighbor}}N_{\text{coil}}$) represents the collapsed k‐space image for slice z, $B_{z}$ (dimension $N_{\text{echo}}N_{k}\times N_{\text{coil}}$) represents the non‐collapsed k‐space image of slice z, and $K_{z}$ (dimension $N_{\text{neighbor}}N_{\text{coil}}\times N_{\text{coil}}$) is the unwrapping kernel. Specifically, the rows of $A_{z}$ and $B_{z}$ concatenate k‐space samples across all echoes, while the columns of $A_{z}$ represent the coil and kernel‐neighbor contributions. We can then solve for the kernel $K_{z}$ using householder rank‐revealing QR decomposition with column‐pivoting [21].

Solving Equation (1) directly will give us a kernel matrix $K_{z}$; however, signals from echoes with long TEs have a lower SNR, and in extreme cases, it could be purely noise. Including these signals while solving for $K_{z}$ may lead to a poor solution. Rather than solving Equation (1) directly, we used matched filtering [22] and weighted matrices $A_{z}$ and $B_{z}$ by the corresponding SNR of k‐space. We define $A_{z}^{\prime }=\left\{a_{i,j}^{\prime }\right\}$ and $B_{z}^{\prime }=\left\{b_{i,j}^{\prime }\right\}$, where $a_{i,j}^{\prime }=a_{i,j}\times \sum_{l=1}^{N_{\text{neighbor}}N_{\text{coil}}}a_{i,l}$ and $b_{i,j}^{\prime }=b_{i,j}\times \sum_{l=1}^{N_{\text{neighbor}}N_{\text{coil}}}a_{i,l}$ and solve the kernel matrix $K_{z}$ according to the equation:

(2) $$\begin{array}{c} A_{z}^{\prime }K_{z}=B_{z}^{\prime } \end{array}$$

This method was developed previously by our group and found to improve SNR compared to when using a single echo for the MB reconstruction and without weighting (Supporting Information).

### 2.4 Image Processing

All preprocessing steps were performed using custom pipelines built with Analysis of Functional NeuroImages (AFNI), FMRIB Software Library (FSL), and Statistical Parametric Mapping 8 (SPM8) tools [23, 24, 25]. Perfusion‐weighted data for both M2‐PCASL acquisitions were derived from the first echo. BOLD data for the low‐resolution M2‐PCASL acquisition was derived from the second echo. ASL data underwent the motion correction procedure outlined by Wang in 2012, where the control‐label acquisition pattern is regressed from the rigid‐body motion estimates [26] while BOLD‐weighted images underwent standard rigid‐body motion correction. Data from each echo timeseries were then smoothed with 3 and 5‐mm FWHM Gaussian kernels which allowed us to compare the effects of smoothing kernel on the parameter estimates. T_1_‐weighted images were brain‐extracted and intensity‐normalized, and transformations to Montreal Neurological Institute (MNI) space were calculated.

### 2.5 Estimation of CBF and CVR

A surround‐subtract technique was used to obtain perfusion‐weighted difference images from the first echo time series of each M2‐PCASL dataset [27]. CBF was calculated in units of mL/100 g/min using the equation [20].

(3) $$\begin{array}{c} \mathrm{CBF}=\frac{6000*\lambda *e^{\frac{\omega }{T_{1\text{blood}}}}}{2*\alpha *T_{1\text{blood}}\left(1-e^{\frac{-\tau }{T_{1\text{blood}}}}\right)}\frac{\Delta S}{S_{0}} \end{array}$$

where $\lambda =\frac{0.9\mathrm{mL}}{g}$ is the brain–blood partition coefficient; ω is the slice‐specific PLD; $T_{1\text{blood}}=1.664\;s$ is the $T_{1}$ of arterial blood at 3T; α=0.85 is the labeling efficiency; τ=1.5088s is the LD; $S_{0}$ is the estimated proton density image (mean control image in our study); ΔS is the perfusion‐weighted difference image. Baseline CBF was obtained by averaging CBF volumes from the baseline period (first 2 min of the scan).

With either CBF or BOLD time series, CVR can be obtained using a voxel‐wise linear regression technique that quantifies the signal dependence on the measured EtCO_2_ in units of %∆signal/mmHg EtCO_2_ [28]. Each voxel time series was temporally smoothed with a moving average filter, and the following model was fit:

(4) $$\begin{array}{c} \mathrm{MRI}\;\text{signal}=\beta_{1}*\mathrm{EtC}O_{2}+\beta_{0}+\varepsilon \end{array}$$

where MRIsignal is either a BOLD or CBF time series, $\mathrm{EtC}O_{2}$ is the mean baseline‐subtracted CO_2_ envelope, and ε is noise. BOLD or CBF CVR is then determined from

(5) $$\begin{array}{c} \;\mathrm{CVR}=\frac{\beta_{1}}{\beta_{0}}. \end{array}$$

Since ASL data are known to be temporally noisy, we also calculated CBF CVR using the equation.

(6) $$\begin{array}{c} \mathrm{CBF}\;\mathrm{CVR}=\frac{\frac{\mathrm{CB}F_{\mathrm{hyp}}-\mathrm{CB}F_{\text{baseline}}}{\mathrm{CB}F_{\text{baseline}}}*100}{\mathrm{EtC}{O_{2}}_{\mathrm{hyp}}-\mathrm{EtC}{O_{2}}_{\text{baseline}}} \end{array}$$

where $\mathrm{CB}F_{\mathrm{hyp}}$ and $\mathrm{CB}F_{\text{baseline}}$ are the average CBF maps during hypercapnia and room air breathing, respectively and $\mathrm{EtC}{O_{2}}_{\mathrm{hyp}}\;\text{and}\;\mathrm{EtC}{O_{2}}_{\text{baseline}}$ are the EtCO_2_ traces during hypercapnia and room air breathing, respectively [29]. This equation reduces the sensitivity of CBF CVR measurements to temporal noise; however, individual fluctuations of EtCO_2_ and their effects on the MR signal are lost.

CBF and CVR were obtained from custom pipelines built in MATLAB R2023a. We excluded 4 subjects from successive analyses due to excessive motion (displacements exceeding 2 mm of translation or 2° of rotation) in either the low or high‐resolution M2‐PCASL datasets or variable EtCO_2_ measurements. Of the 9 remaining subjects, 4 had a LD of 1.5 s.

### 2.6 Statistical Analyses

Reliability was quantified using the intraclass correlation coefficient (ICC), a correlation metric commonly used for assessing reliability in test–retest studies. We used the ICC(2,1) definition which penalizes systematic differences in measurements between sessions [30]. From the low‐resolution M2‐PCASL data, ICCs were calculated for baseline CBF, CBF CVR, and BOLD CVR. From the high‐resolution M2‐PCASL data, ICCs were calculated only for baseline CBF since this scan lacked a gas challenge. We computed ICCs voxel‐wise and within regions of interest (ROIs) using custom scripts written in Python 3.11.9. ROIs from the Automated Anatomical Labeling (AAL) atlas [31] were aligned to the subjects' native space to minimize interpolation errors. Besides AAL atlas ROIs, we included gray and white matter (GM and WM) which were obtained from the subjects' anatomical image and co‐registered to the M2‐PCASL images.

For the reliability analysis of baseline CBF, we combined subjects scanned with different LDs into a single dataset for each acquisition to maintain a reasonable sample size. Before proceeding with the analysis, we tested for potential confounding effects of the LD on the baseline CBF estimates by constructing separate linear mixed effects models for each acquisition where LD was modeled as a fixed effect, subjects were modeled as random effects, and mean baseline CBF was the outcome.

### 2.7 Assessing Factors Influencing CBF tSNR

To elucidate sources of variability in the CBF time courses, we constructed linear mixed effects models to evaluate the effects of motion, MB factor/image resolution, and LD on repeated CBF tSNR measurements. CBF tSNR was calculated from the baseline period of the time course during room air inhalation. The degree of motion was quantified using framewise displacement (FD) which was calculated from the sum of absolute differences of translations and rotations in the x, y, and z planes. Rotations were converted from degrees to millimeters, assuming a head radius of 50 mm. These analyses were performed in R 4.4.1.

### 2.8 Assessing the Effects of CO_2_ Concentration on BOLD Signal and CVR

To determine the effects of CO_2_ concentration on the MR signal and CVR estimates, we compared two subject groups who underwent a gas challenge with either 5% or 8% CO_2_ during the low‐resolution M2‐PCASL acquisition. The 8% CO_2_ group consisted of subjects from the first run of the test–retest study. The 5% CO_2_ group consisted of nine healthy subjects from a prior study from our lab (age = 28.1 ± 4.3 years, 3 males). We compared CVR and temporal CNR (tCNR) of BOLD data between groups using independent samples and non‐parametric *t*‐tests to account for our small sample size and potential normality violations. Statistical significance was determined using an alpha level of 0.05. tCNR was calculated using the equation:

(7) $$\begin{array}{c} \text{tCNR}=\frac{{\bar{x}}_{CO_{2}}-{\bar{x}}_{\text{baseline}}}{\sigma_{\text{baseline}}} \end{array}$$

where ${\bar{x}}_{CO_{2}}$ is the mean BOLD signal during hypercapnia, ${\bar{x}}_{\text{baseline}}$ is the mean BOLD signal during room air inhalation, and $\sigma_{\text{baseline}}$ is the standard deviation of the signal during room air inhalation.

## 3 Results

### 3.1 Reliability of Baseline CBF in the Low and High‐Resolution M2‐PCASL Acquisitions

**FIGURE 2:**
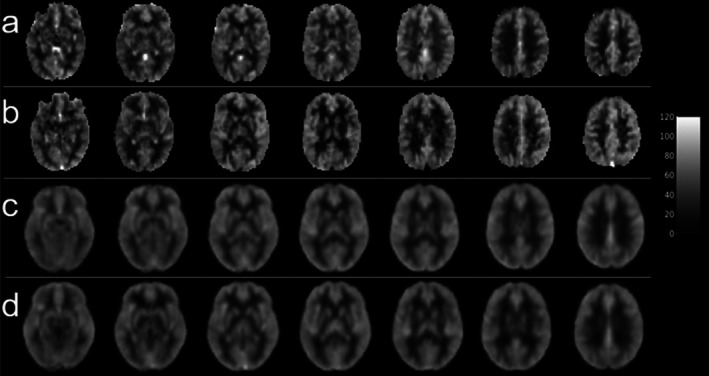
Baseline cerebral blood flow (CBF) maps generated from smoothing M2‐PCASL data with a 5‐mm FWHM Gaussian kernel. Representative low‐resolution (a) and high‐resolution (b) baseline CBF maps from a single subject. Group mean baseline CBF maps from the low‐resolution (c) and high‐resolution (d) acquisitions.

We found no significant effect of LD on the mean baseline CBF estimates from either sequence, so we included data from both LDs in our analyses to increase the sample size. Representative and group average baseline CBF maps generated from the 5‐mm FWHM Gaussian kernel are shown in Figure 2; the low‐resolution acquisition had an average GM and WM baseline CBF of 51.37 ± 12.05 and 43.81 ± 9.87 mL/100 g/min, respectively, and the high‐resolution acquisition had an average GM and WM baseline CBF of 51.30 ± 14.17 and 41.74 ± 10.24 mL/100 g/min, respectively.

**FIGURE 3:**
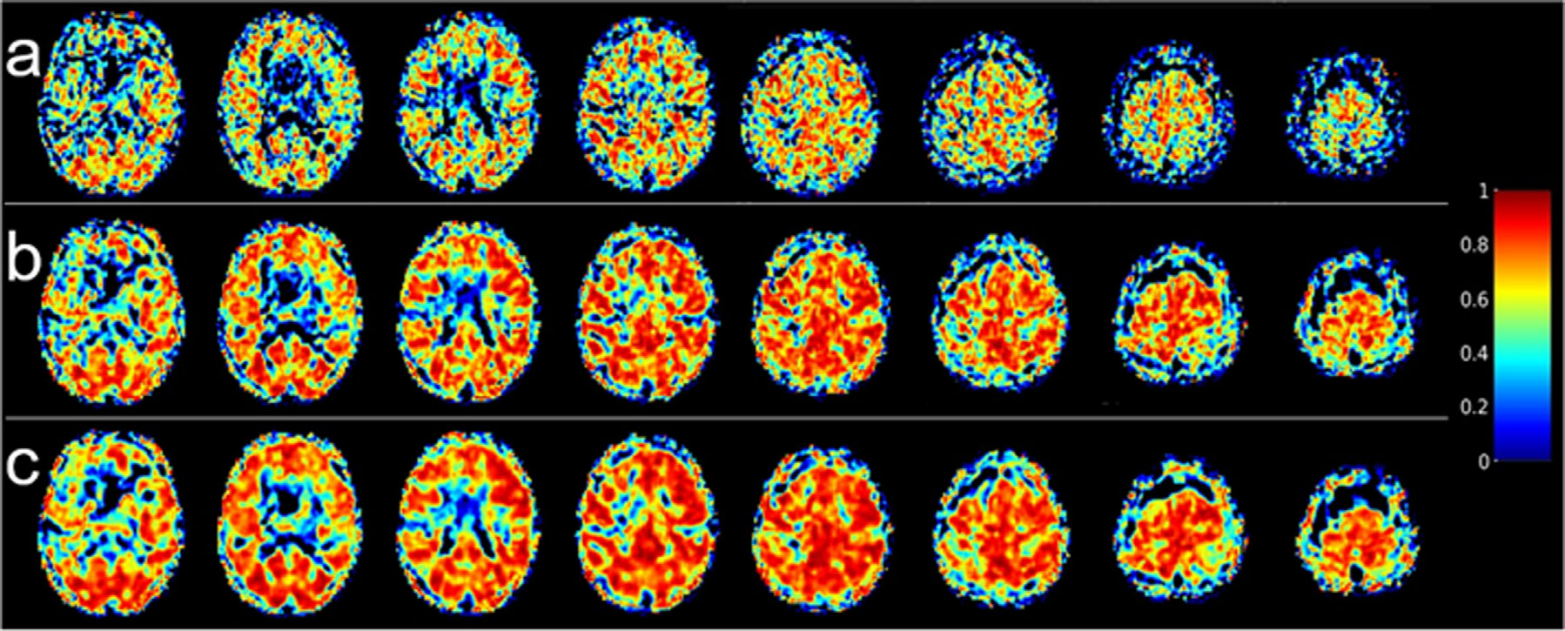
Voxel‐wise intraclass correlation coefficient (ICC) maps obtained from low‐resolution baseline cerebral blood flow images generated from smoothing motion‐corrected M2‐PCASL data with different Gaussian kernels prior to control‐label subtraction: (a) no smoothing; (b) FWHM = 3 mm; (c) FWHM = 5 mm.

**FIGURE 4:**
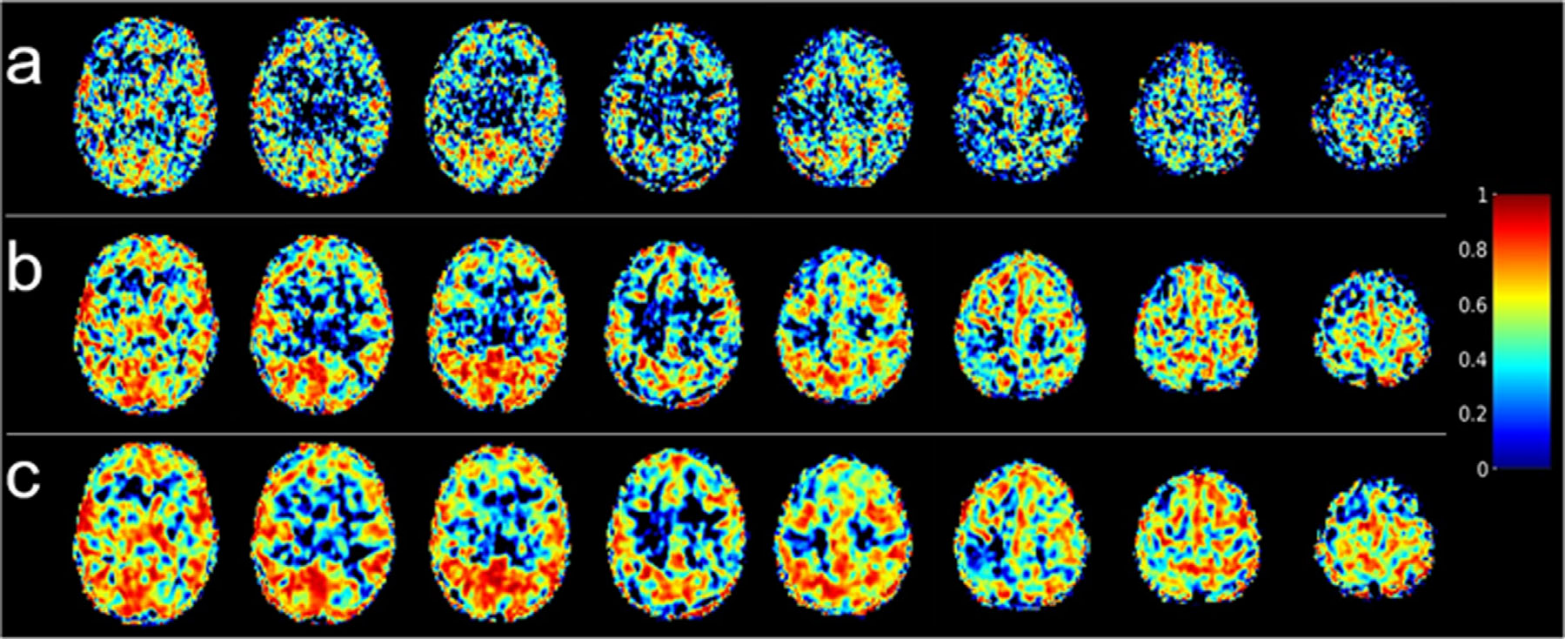
Voxel‐wise intraclass correlation coefficient (ICC) maps obtained from high‐resolution baseline cerebral blood flow images generated from smoothing motion‐corrected M2‐PCASL data with different Gaussian kernels prior to control‐label subtraction: (a) no smoothing; (b) FWHM = 3 mm; (c) FWHM = 5 mm.

Voxel‐wise ICC maps for baseline CBF estimates obtained from the low and high‐resolution M2‐PCASL acquisitions are shown in Figures 3 and 4, respectively. Increased smoothing increased voxel‐wise reliability for both acquisitions. For the low‐resolution scan, mean voxel‐wise ICCs in the GM were 0.42 ± 0.17, 0.52 ± 0.19, and 0.56 ± 0.20 for unsmoothed, 3‐mm, and 5‐mm kernels, respectively. Corresponding values for the high‐resolution scan were 0.32 ± 0.14, 0.45 ± 0.16, and 0.51 ± 0.18.

**FIGURE 5:**
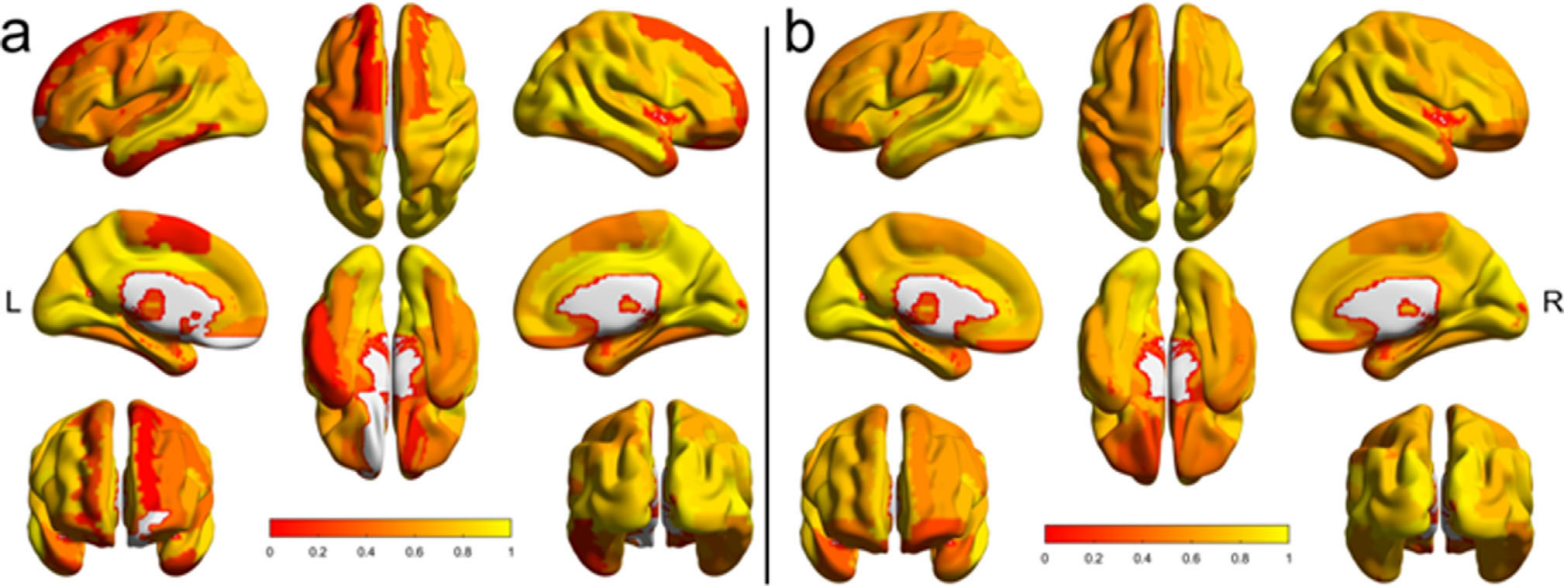
Surface renderings of intraclass correlation coefficients for baseline cerebral blood flow (CBF) calculated in regions of interest defined by the Automated Anatomical Labeling (AAL) atlas in the low‐resolution (a) and high‐resolution (b) M2‐PCASL acquisitions. Baseline CBF maps were generated from smoothing motion‐corrected M2‐PCASL data with a 5‐mm FWHM Gaussian kernel.

For the ROI analysis, we included all AAL atlas ROIs from which CBF could be estimated. For example, cerebellar estimates in some subjects were unstable; hence, the cerebellum was excluded in this analysis. Results for the ROI reliability analysis of ASL data smoothed with a 5‐mm FWHM Gaussian kernel are shown in Figure 5. For both M2‐PCASL acquisitions, most ROIs achieve good to excellent reliability with an average ICC across AAL ROIs of 0.62 ± 0.27 and 0.69 ± 0.19 in the low and high‐resolution acquisitions, respectively. Additionally, the GM, WM, and whole brain had ICCs exceeding 0.88. Corresponding surface renderings for the unsmoothed and 3‐mm FWHM smoothed data can be found in the Supporting Information, along with tables reporting ROI ICCs. Without smoothing and smoothing with a 3‐mm FWHM kernel, the average ICC across AAL ROIs was 0.48 ± 0.35 and 0.58 ± 0.29, respectively, for the low‐resolution acquisition and 0.62 ± 0.24 and 0.67 ± 0.21, respectively, for the high‐resolution acquisition. Like the voxel‐wise results, increased smoothing increased ROI reliability.

### 3.2 Reliability of BOLD CVR

**FIGURE 6:**
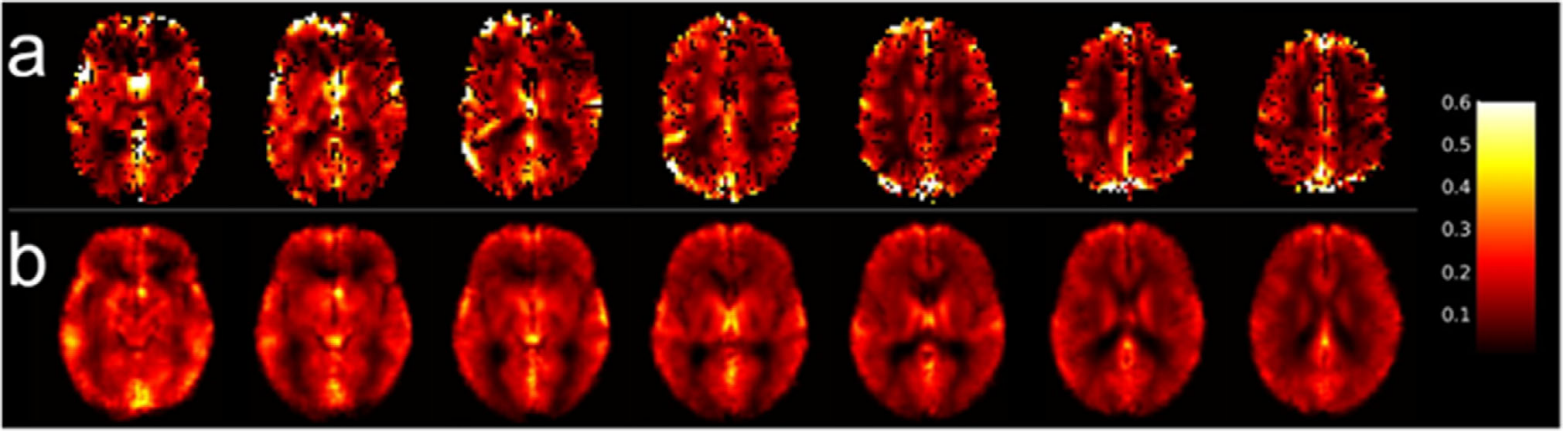
Blood oxygen level‐dependent (BOLD) cerebrovascular reactivity (CVR) maps obtained from smoothing BOLD data with a 5‐mm FWHM Gaussian kernel: (a) representative BOLD CVR map from a single subject; (b) group average BOLD CVR map. Units are %∆BOLD/mmHg EtCO_2_.

Representative and group average BOLD CVR maps generated from the 5‐mm FWHM kernel are shown in Figure 6; the average CVR in GM and WM was 0.22 ± 0.05 and 0.16 ± 0.03 %∆BOLD/mmHg EtCO_2_, respectively.

**FIGURE 7:**
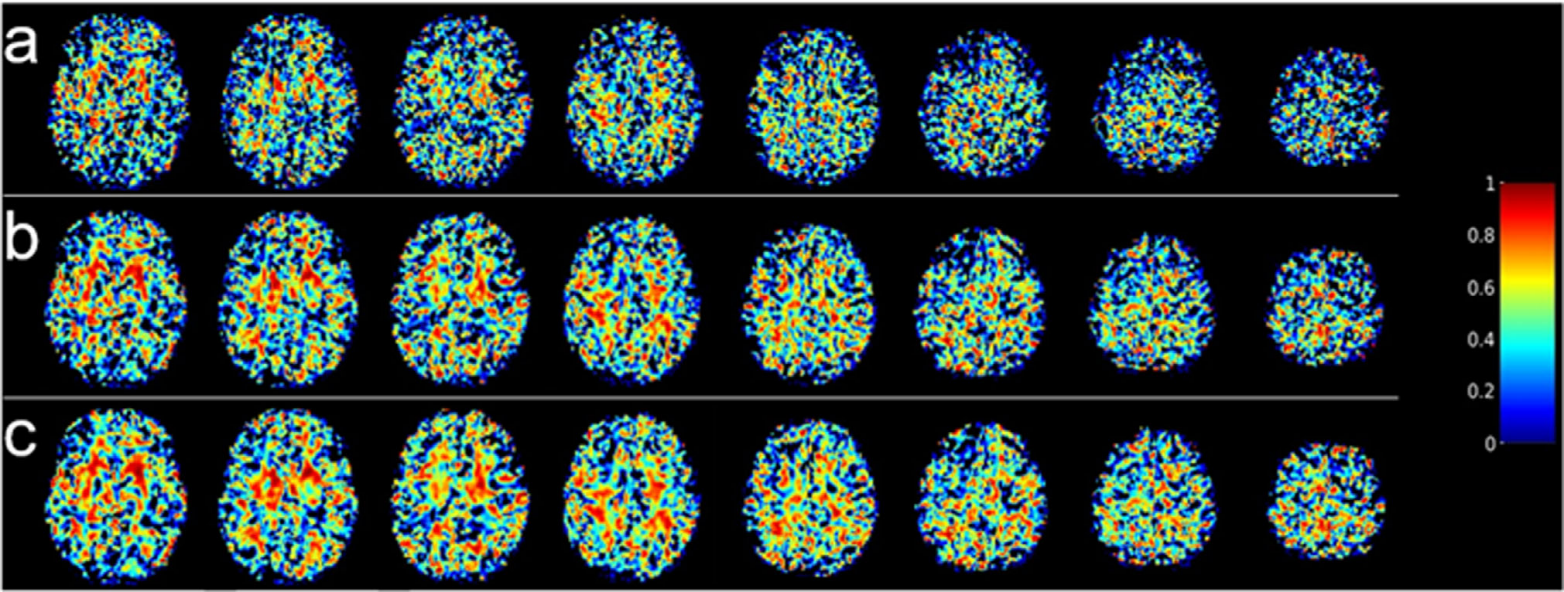
Voxel‐wise intraclass correlation coefficient (ICC) maps obtained from blood oxygen level‐dependent (BOLD) cerebrovascular reactivity (CVR) maps. BOLD data smoothed with different Gaussian kernels prior to CVR computation: (a) no smoothing; (b) FWHM = 3 mm; (c) FWHM = 5 mm.

Voxel‐wise ICC maps for BOLD CVR are shown in Figure 7. Voxel‐wise reliability increased with increased smoothing. In the GM, mean ICCs obtained were 0.23 ± 0.14 with no smoothing, 0.31 ± 0.15 for 3‐mm FWHM smoothing, and 0.35 ± 0.15 for 5‐mm FWHM smoothing. Overall, voxel‐wise reliability was fair across smoothing kernels.

**FIGURE 8:**
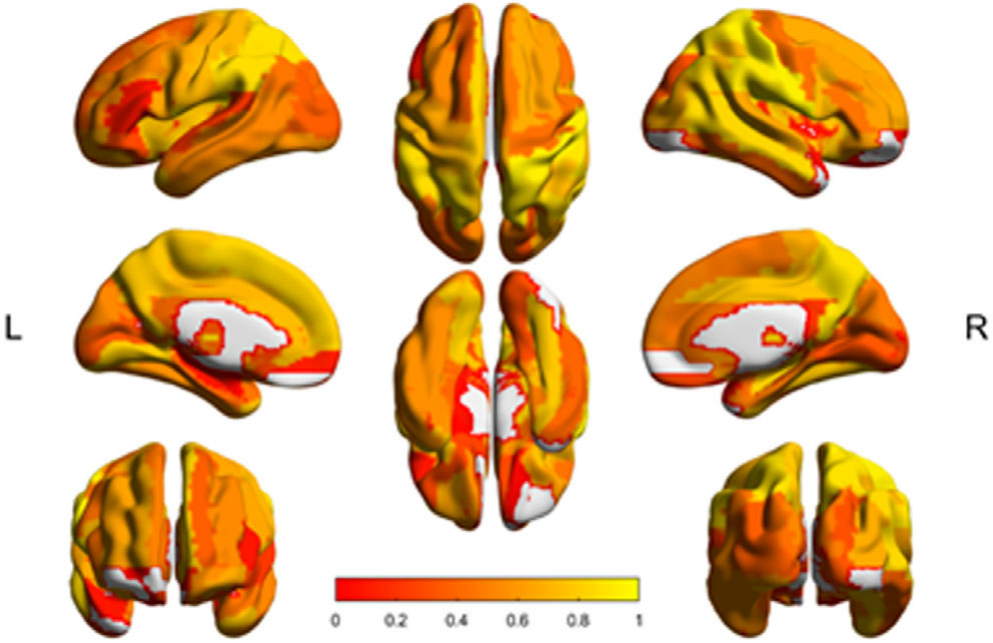
Surface renderings of intraclass correlation coefficients for blood oxygen level‐dependent (BOLD) cerebrovascular reactivity (CVR) calculated in regions of interest defined by the Automated Anatomical Labeling (AAL) atlas. BOLD CVR obtained from BOLD images smoothed with 5‐mm FWHM Gaussian kernel.

Figure 8 shows a surface rendering of BOLD CVR ICCs within the AAL ROIs after smoothing BOLD data with a 5‐mm FWHM Gaussian kernel. With this kernel, most ROIs exhibit moderate to excellent reliability with an average ICC across all ROIs of 0.54 ± 0.29. Moreover, the total GM exhibited good reliability with an ICC of 0.84 (95% confidence interval (CI): 0.43–0.96). Corresponding surface renderings for the unsmoothed and 3‐mm FWHM smoothed data can be found in the Supporting Information along with tables reporting ROI ICCs. Like the voxel‐wise analysis, the average ROI ICC increased with increased smoothing. Without smoothing and smoothing with a 3‐mm FWHM kernel, the average ICCs were 0.37 ± 0.34 and 0.50 ± 0.30, respectively. However, ROIs from the subjects' anatomical images (GM, WM, and whole brain) were slightly higher in the 3‐mm FWHM smoothed data compared to the 5‐mm FWHM smoothed data. For example, the GM ICC in the 3‐mm FWHM smoothed data was 0.86 (95% CI: 0.50–0.97) while the GM ICC in the 5‐mm FWHM smoothed data was 0.84 (95% CI: 0.43–0.96).

### 3.3 Reliability of CBF CVR

**FIGURE 9:**
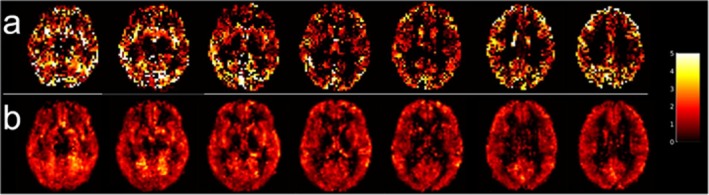
Cerebral blood flow (CBF) cerebrovascular reactivity (CVR) maps from M2‐PCASL data smoothed with a 5‐mm FWHM Gaussian kernel: (a) representative subject; (b) group average. Units are %∆CBF/mmHg EtCO_2_. CVR calculated from Equation (6).

The poor CBF tSNR prohibited us from obtaining reliable estimates of CBF CVR using linear regression. An example of the GM CBF time series is shown in the Supporting Information. Unlike BOLD signal which produces a robust response to hypercapnia, CBF is more variable, yielding unstable estimates for CVR. We were able to obtain CVR using Equation (6), but only after excluding outliers outside the central 96% of the intensity distribution (Figure 9). Without smoothing, the GM and WM ICCs were unreliable (ICC < 0). Smoothing ASL data with the 3‐mm FWHM Gaussian kernel, the GM and WM ICCs were 0.42 (95% CI: −0.36–0.84) and 0.33 (95% CI: −0.46–0.80), respectively, and smoothing with the 5‐mm FWHM Gaussian kernel, the GM and WM ICCs were 0.55 (95% CI: −0.17–0.88) and 0.47 (95% CI: −0.27–0.85), respectively. Voxel‐wise estimates did not have sufficient SNR to determine reliability, and smaller ROIs from the AAL atlas were mostly unreliable and not reported.

### 3.4 Factors Influencing CBF tSNR

Before fitting the linear mixed effects models, we observed greater motion in the low‐resolution acquisition compared to the high‐resolution acquisition (*p* = 0.0005 in the second run, *p* = 0.055 in the first run). This necessitated separate analyses to prevent motion from confounding our interpretation of sequence‐related effects. Table 1 shows models for each acquisition using the 5‐mm FWHM smoothed data. LD did not affect the CBF tSNR in either dataset (*p* > 0.53). Higher average and maximum FD were significantly associated with lower tSNR (*p* < 0.002). Although the low‐resolution data had a slightly higher whole‐brain tSNR than the high‐resolution data (2.04 ± 0.72 vs. 1.59 ± 0.53, *p* < 0.03), estimates for each factor (LD, average FD, and maximum FD) were comparable across acquisitions. Results for the unsmoothed and 3‐mm FWHM smoothed data were similar to the 5‐mm FWHM smoothed data and can be found in the Supporting Information.

**TABLE 1:** Linear mixed effects models evaluating the factors influencing the temporal SNR (tSNR) of the low and high‐resolution M2‐PCASL baseline cerebral blood flow (CBF) time courses obtained from smoothing the M2‐PCASL data with a 5‐mm FWHM Gaussian filter. FD stands for framewise displacement.

Factors influencing baseline CBF tSNR (5‐mm FWHM smoothed)			
Outcome	Labeling duration	Average FD	Maximum FD
Low‐resolution baseline CBF tSNR	0.08 (*p* = 0.88)	NA	NA
	NA	**−7.53 (*p* = 8e‐05)**	NA
	NA	NA	**−1.07 (*p* = 0.0008)**
High‐resolution baseline CBF tSNR	0.08 (*p* = 0.53)	NA	NA
	NA	**−6.54 (*p* = 0.002)**	NA
	NA	NA	**−1.08 (*p* = 0.001)**

*Note*: Bold font indicates statistical significance.

### 3.5 Effects of CO_2_ Concentration on BOLD Signal and CVR

Group comparisons of BOLD CVR and tCNR between the 5% and 8% CO_2_ groups were performed for unsmoothed and smoothed BOLD data. No significant differences were observed in CVR or tCNR in the GM, WM, or whole brain as assessed by independent samples *t*‐tests and Wilcoxon rank tests; although, the tCNR in the 8% CO_2_ group was always higher and less variable compared to the 5% group. Moreover, these group differences became more pronounced with increased BOLD smoothing. Specifically, the difference in whole‐brain tCNR between the 5% and 8% groups rose from 0.68 (unsmoothed), to 1.22 (3‐mm FWHM), and 1.61 (5‐mm FWHM). The largest difference in the 5‐mm smoothed data trended toward significance (*p* = 0.09). Detailed results can be found in the Supporting Information.

## 4 Discussion

We demonstrate that M2‐PCASL combined with a hypercapnia challenge provides a comprehensive, reliable hemodynamic assessment with improved spatial and temporal resolutions relative to existing methods. At the time of our initial abstract, another group was also working toward developing a MB multi‐echo pseudo‐continuous ASL (PCASL) sequence for CVR assessment [32]. With this sequence, they assessed the test–retest reliability of BOLD and CBF CVR using the breath‐hold (BH) technique. While BH techniques have several limitations, including greater inter‐ and intra‐subject variability and the inability to provide quantitative CVR measurements, which prevents standardized comparisons, they may be more accessible than gas‐inhalation techniques when specialized equipment is unavailable. Nonetheless, gas‐inhalation techniques are widely used to reliably and quantitatively measure CVR [33]. Previous studies have investigated the test–retest reliability of CVR using the 5% CO_2_ stimulus [17, 29, 34] as well as BH techniques [32]; however, to our knowledge, this is the first study to evaluate the test–retest reliability of CBF and CVR derived from a MB protocol combined with a gas‐based hypercapnia challenge and concurrent EtCO_2_ measurement. Additionally, we used the less common 8% CO_2_ stimulus to understand its effects on CVR reliability and to aid future studies when selecting which stimulus to use.

### 4.1 Baseline CBF Derived From Low and High‐Resolution M2‐PCASL Acquisitions

Baseline CBF estimates from both M2‐PCASL acquisitions had ICC values comparable or superior to what is reported in literature [35, 36, 37] with excellent reliability in GM, WM, and whole brain regions. Frontal regions exhibited lower reliability and asymmetry across hemispheres which could be attributed to signal distortion artifacts common in EPI sequences. Increased smoothing increased sensitivity and improved reliability of baseline CBF estimates in both acquisitions while spatial specificity decreased. Average GM and WM baseline CBF estimates remained relatively stable across both smoothing kernels and image resolutions, ranging from 51 to 52 mL/100 g/min in the GM and 39 to 44 mL/100 g/min in the WM. Moreover, the low‐resolution baseline CBF estimates may be better in terms of voxel‐wise reliability but worse in terms of ROI reliability compared to the high‐resolution estimates. This may be explained by differences in sampling and image registration procedures between the reliability analyses. Also, the low‐resolution data is higher in SNR than the high‐resolution data and thus may exhibit higher voxel‐wise reliability, but the high‐resolution data contains more voxels per ROI and will experience an SNR gain when many voxels are averaged, thus increasing the ROI reliability.

In either M2‐PCASL dataset, we did not notice an effect of LD on the tSNR of the baseline CBF time courses. Our sample size is small and might be insufficient to capture the small effects of LD on the CBF tSNR. We did, however, see negative effects of average and maximum FD on the CBF tSNR with average FD having the greater effect. The increased motion in the low‐resolution acquisition is likely because the subjects were breathing through a mouthpiece during this acquisition which may have been uncomfortable, resulting in increased motion. We also did not randomize the order of the acquisitions, which may have contributed to systematic differences in movement.

The primary reason that the temporal feature of BOLD signal was not investigated in this study is because CBF is inherently noisier than BOLD and understanding the factors influencing the tSNR of the CBF data allows for potential SNR optimization strategies to be developed. Given the extensive scope of this study, we constrained our focus to CBF where SNR optimization is most crucial.

In terms of reliability, optimizing the CBF tSNR data might be more important when estimating CBF CVR than when estimating baseline CBF. Although the low‐resolution acquisition had a slightly higher CBF tSNR than the high‐resolution acquisition (2.04 ± 0.72 vs. 1.59 ± 0.53), this did not always translate to improved reliability of baseline CBF estimates. Since baseline CBF is computed by averaging over many time points, the random noise in the time course is effectively smoothed, reducing the impact of the tSNR on the reliability of baseline CBF estimates. When CBF CVR is computed using linear regression, each time point may differentially affect the final CVR estimate. For this reason, noise and extreme outliers may have a greater impact on the reliability and accuracy of CVR estimates from linear regression.

### 4.2 Reliability of CVR Obtained From the Low‐Resolution M2‐PCASL Acquisition

Voxel‐wise and ROI reliability of BOLD CVR from our approach was also comparable to reports in the literature. For example, Cohen and Wang who used the BH technique to estimate CVR, reported an average voxel‐wise GM ICC of 0.44; however, they employed the ICC (3,1) definition which assesses consistency rather than absolute agreement as in the ICC(2,1) definition [32]. While either definition can be justified for this task, the ICC(2,1) definition is more conservative and is always less than the ICC(3,1) definition. Additionally, the GM and WM BOLD CVR ICC values obtained here (0.84 and 0.81) were comparable to Kassner et al. (0.81 and 0.66) who also used an 8% CO_2_ gas challenge [38].

We observed that the whole brain, GM, and WM BOLD CVR ICCs peaked in the 3‐mm FWHM smoothed data while the average ICC across the AAL ROIs peaked in the 5‐mm FWHM smoothed data; however, the differences were quite small. The discrepancy likely arises because the GM, WM, and whole brain ROIs are derived from the subjects' anatomy while the AAL ROIs are from an atlas and transformed to the subjects' space. As BOLD signal is relatively high in tSNR, the 3‐mm FWHM kernel may offer sufficient sensitivity. Irrespective of smoothing, ICCs were lower in the frontal and temporal lobes, consistent with the known EPI signal dropout in these areas. For the voxel‐wise analysis, we observed increased reliability with increased BOLD smoothing. Spatially specific parameter estimates could be preserved in small regions such as the hippocampus by using less spatial smoothing.

We acknowledge the potential presence of control‐label modulation in the BOLD‐weighted data. We attempted to remove this modulation from the BOLD‐weighted data as done for the ASL data but did not observe improvements in tSNR or reliability relative to standard motion correction. The lower SNR in the second echo might make estimating and removing the modulation pattern more difficult compared to the first echo. Consequently, standard motion correction was used in the BOLD‐weighted data.

The multi‐echo combination procedure described by Cohen and Wang was also attempted [32]. This procedure weights each echo by a function of T_2_*, which is fit from an exponential equation. The weighted combination of echoes was found to improve reliability and SNR of BOLD CVR estimates [32]. In our dual‐echo acquisition, we cannot reliably compute these weights as the best estimate of T_2_* we can make is based on a linear fit. For this reason, we analyzed each echo separately.

The inability of the low‐resolution M2‐PCASL acquisition to reliably estimate CBF CVR using linear regression can be attributed to a few factors. One factor is the lack of background suppression (BS). Ghariq et al. showed that BS can markedly improve the SNR of the ASL signal while only slightly degrading the SNR of the BOLD signal, justifying its use in multi‐echo ASL/BOLD acquisitions [39]. Increased head motion during the acquisition with the gas challenge compared to the acquisition without the gas challenge seems to have had an adverse effect on the CBF tSNR as shown in our linear mixed effects models. Equation (6) provides an alternative means to compute CVR from CBF data, albeit the results are still noisy and less reproducible than BOLD CVR. Previous studies have also reported that CBF CVR is less reliable than BOLD CVR [32, 34].

EtCO_2_ measurements can be noisy and vary significantly between subjects and runs, potentially degrading the reliability of CVR. In developing our processing pipelines, we tried smoothing EtCO_2_ measurements to varying degrees before deciding on the current smoothing kernel which produced the best reliability. Individual CO_2_ fluctuations are usually much smaller in magnitude than the increase due to hypercapnia; thus, CVR measurements should largely reflect the hypercapnic response.

### 4.3 Effects of CO_2_ Concentration on CVR Estimation

For fixed inspired CO_2_ challenges, 5% CO_2_ is the most widely used concentration in literature, and few studies have investigated other concentrations for measuring CVR. Theoretically, higher concentrations of inspired CO_2_ will yield higher concentrations of expired CO_2_ (a surrogate for arterial CO_2_); although, the extent of increase varies across individuals due to differences in breathing rate, lung function, and breathing depth. While it is ideal to use higher arterial CO_2_ concentrations to induce greater increases in CBF, it is unclear whether higher CO_2_ concentrations beyond 5% would provide a sufficient increase in tCNR and sensitivity to justify their use [40]. We exploited the 8% CO_2_ stimulus in this study to address this ambiguity.

We observed no significant differences in BOLD CVR or tCNR between 5% and 8% CO_2_ groups; however, compared to the 5% group, the 8% group exhibited numerically higher and less variable tCNR in the GM, WM, and whole brain. Reduced variability in the 8% CO_2_ group suggests that reliability of CVR may be improved using 8% CO_2_, although this should be confirmed with repeated acquisitions for 5% CO_2_. The differences in tCNR between the groups did not seem to affect the measured CVR, indicating that further gains in tCNR might not meaningfully enhance the sensitivity or reliability of CVR estimates. Higher CO_2_ concentrations may have disadvantages including increased subject motion and discomfort and population exclusion (e.g., elderly, diseased) that could offset sensitivity or tCNR gains. Compared to acquisitions without a gas challenge, both the 5% and 8% CO_2_ groups had increased motion, but there were no differences in motion between these groups.

### 4.4 Limitations

This study is limited by the small sample size which reduced our power to detect LD or CO_2_ concentration effects. tSNR was constrained by the inherently low signal of ASL and the lack of BS; acquiring additional hypercapnic blocks could improve future CVR calculations. Moreover, the lack of standardization in processing ASL/BOLD data makes our results pipeline‐dependent. Finally, this study was limited to young, healthy participants, which may restrict the generalizability of our findings. Specifically, the comparison of BOLD CVR between the 5% and 8% CO_2_ groups may not extend to elderly or diseased populations. While our reliability analyses support the utility of our methods for assessing cerebrovascular parameters in young, healthy individuals, future studies are needed to validate their applicability in clinical and aging populations.

## 5 Conclusion

We demonstrate that our M2‐PCASL sequence with hypercapnia reliably estimates hemodynamic parameters at improved spatial and temporal resolutions relative to existing methods and can simultaneously acquire multiple hemodynamic parameters, offering the potential to noninvasively assess cerebrovascular health in aging and diseased populations. Sources of variability including subject motion can differentially impact each parameter and should be carefully accounted for. Future studies should confirm the CO_2_ concentration for CVR estimation that is optimal for subject safety and reliability.

## Funding

This work was supported by the National Institutes of Health (1R01 AG089806, P30AG066511, R01AG070937, and R01AG072603).

## Conflicts of Interest

Dr. Qiu reports grants from National Institutes of Health during the conduct of the study and non‐financial support from Siemens Healthcare outside of the submitted work.

## Supporting information


**Figure S1:** A comparison of different multi‐band multi‐echo image reconstruction methods. These images demonstrate the reconstruction results of one slice of the second echo acquisition. The first row is the comparison between computing kernel matrix using only the first echo and using all the echoes. Note that neither of them weighted k‐space signal during reconstruction. The red circle indicates the location of the artifact. The fourth image on the first row is the same as the first image but with a different color display range to emphasize the location of the artifact. The second row is the comparison between computing kernel matrix between with k‐space weighting and without k‐space weighting.
**Figure S2:** Surface renderings of intraclass correlation coefficients for baseline cerebral blood flow (CBF) calculated in regions of interest defined by the Automated Anatomical Labeling (AAL) atlas. Results are shown for the low‐resolution M2‐PCASL data: (a) unsmoothed data; (b) data smoothed with a 3‐mm FWHM Gaussian kernel.
**Figure S3:** Surface renderings of intraclass correlation coefficients for baseline cerebral blood flow (CBF) calculated in regions of interest defined by the Automated Anatomical Labeling (AAL) atlas. Results are shown for the high‐resolution M2‐PCASL data: (a) unsmoothed data; (b) data smoothed with a 3‐mm FWHM Gaussian kernel.
**Figure S4:** Surface renderings of intraclass correlation coefficients for blood oxygen level‐dependent (BOLD) cerebrovascular reactivity (CVR) calculated in regions of interest defined by the Automated Anatomical Labeling (AAL) atlas: (a) no BOLD smoothing; (b) BOLD smoothing with a 3‐mm FWHM Gaussian kernel.
**Figure S5:** Average gray matter blood oxygen level‐dependent (BOLD) and cerebral blood flow (CBF) signal with temporal smoothing during hypercapnia in a representative subject. In this figure, BOLD data is not spatially smoothed while the control‐label pairs from the arterial spin labeled data is smoothed with a 5‐mm FWHM Gaussian kernel.
**Table S1:** Intraclass correlation coefficients (ICCs) and 95% confidence intervals (CIs) computed from average baseline cerebral blood flow (CBF) estimates in the regions of interest (ROIs) provided by the Automated Anatomical Labeling (AAL) atlas. Results are shown for the low‐resolution M2‐PCASL data with no smoothing and with smoothing with 3 and 5‐mm FWHM Gaussian kernels. Abbreviations: L: left, R: right, Ant: Anterior, Mid: middle, Post: posterior, Oper: operculum, Orb: orbital, Tri: triangularis, Sup: superior, GM: gray matter, WM: white matter.
**Table S2:** Intraclass correlation coefficients (ICCs) and 95% confidence intervals (CIs) computed from average baseline cerebral blood flow (CBF) estimates in the regions of interest (ROIs) provided by the Automated Anatomical Labeling (AAL) atlas. Results are shown for the high‐resolution M2‐PCASL data with no smoothing and smoothing with 3 and 5‐mm FWHM Gaussian kernels. Abbreviations: L: left, R: right, Ant: Anterior, Mid: middle, Post: posterior, Oper: operculum, Orb: orbital, Tri: triangularis, Sup: superior, GM: gray matter, WM: white matter.
**Table S3:** Intraclass correlation coefficients (ICCs) and 95% confidence intervals (CIs) computed from average blood oxygen level‐dependent (BOLD) cerebrovascular reactivity (CVR) estimates in the regions of interest (ROIs) provided by the Automated Anatomical Labeling (AAL) atlas. Results are shown for the low‐resolution M2‐PCASL data with no smoothing and smoothing with 3 and 5‐mm FWHM Gaussian kernels. Abbreviations: L: left, R: right, Ant: Anterior, Mid: middle, Post: posterior, Oper: operculum, Orb: orbital, Tri: triangularis, Sup: superior, GM: gray matter, WM: white matter.
**Table S4:** Comparisons of blood oxygen level‐dependent (BOLD) cerebrovascular reactivity (CVR) and temporal contrast to noise ratio (tCNR) between 5% and 8% inspired carbon dioxide (CO_2_) studies in gray matter (GM), white matter (WM), and whole brain regions of interest (ROIs). No BOLD smoothing.
**Table S5:** Comparisons of blood oxygen level‐dependent (BOLD) cerebrovascular reactivity (CVR) and temporal contrast to noise ratio (CNR) between 5% and 8% inspired carbon dioxide (CO_2_) studies in gray matter (GM), white matter (WM), and whole brain regions of interest (ROIs). BOLD data smoothed with a 3‐mm FWHM Gaussian filter.
**Table S6:** Comparisons of blood oxygen level‐dependent (BOLD) cerebrovascular reactivity (CVR) and temporal contrast to noise ratio (CNR) between 5% and 8% inspired carbon dioxide (CO_2_) studies in gray matter (GM), white matter (WM), and whole brain regions of interest (ROIs). BOLD data smoothed with a 5‐mm FWHM Gaussian filter.
**Table S7:** Linear mixed effects models evaluating the factors influencing the temporal SNR (tSNR) of the low and high‐resolution M2‐PCASL baseline cerebral blood flow (CBF) time courses obtained from the unsmoothed M2‐PCASL data. FD stands for framewise displacement. Bold font indicates statistical significance.
**Table S8:** Linear mixed effects models evaluating the factors influencing the temporal SNR (tSNR) of the low and high‐resolution M2‐PCASL baseline cerebral blood flow (CBF) time courses obtained from smoothing the M2‐PCASL data with a 3‐mm FWHM Gaussian filter. FD stands for framewise displacement. Bold font indicates statistical significance.

## Data Availability

The data that support the findings of this study are available from the corresponding author upon reasonable request.
