# Supplementary material for: Simultaneous Cerebral Blood Flow and Cerebrovascular Reactivity Obtained From Novel Multi‐Band Multi‐Echo Pseudo‐Continuous Arterial Spin Labeling (M2‐PCASL) Sequence: A Test–Retest Reliability Study
Source: Magn Reson Med. 2026 Feb 18;96(1):37–48. doi: 10.1002/mrm.70307 (PMC13156436; doi:10.1002/mrm.70307)
Supplement: Supplementary file 1 — Figure S1: A comparison of different multi‐band multi‐echo image reconstruction methods. These images demonstrate the reconstruction results of one slice of the second echo acquisition. The first row is the comparison between computing kernel matrix using only the first echo and using all the echoes. Note that neither of them weighted k‐space signal during reconstruction. The red circle indicates the location of the artifact. The fourth image on the first row is the same as the first image but with a different color display range to emphasize the location of the artifact. The second row is the comparison between computing kernel matrix between with k‐space weighting and without k‐space weighting. Figure S2: Surface renderings of intraclass correlation coefficients for baseline cerebral blood flow (CBF) calculated in regions of interest defined by the Automated Anatomical Labeling (AAL) atlas. Results are shown for the low‐resolution M2‐PCASL data: (a) unsmoothed data; (b) data smoothed with a 3‐mm FWHM Gaussian kernel. Figure S3: Surface renderings of intraclass correlation coefficients for baseline cerebral blood flow (CBF) calculated in regions of interest defined by the Automated Anatomical Labeling (AAL) atlas. Results are shown for the high‐resolution M2‐PCASL data: (a) unsmoothed data; (b) data smoothed with a 3‐mm FWHM Gaussian kernel. Figure S4: Surface renderings of intraclass correlation coefficients for blood oxygen level‐dependent (BOLD) cerebrovascular reactivity (CVR) calculated in regions of interest defined by the Automated Anatomical Labeling (AAL) atlas: (a) no BOLD smoothing; (b) BOLD smoothing with a 3‐mm FWHM Gaussian kernel. Figure S5: Average gray matter blood oxygen level‐dependent (BOLD) and cerebral blood flow (CBF) signal with temporal smoothing during hypercapnia in a representative subject. In this figure, BOLD data is not spatially smoothed while the control‐label pairs from the arterial spin labeled data is smoothed with a 5‐mm FWHM Ga [file MRM-96-37-s001.docx]

**Figures**


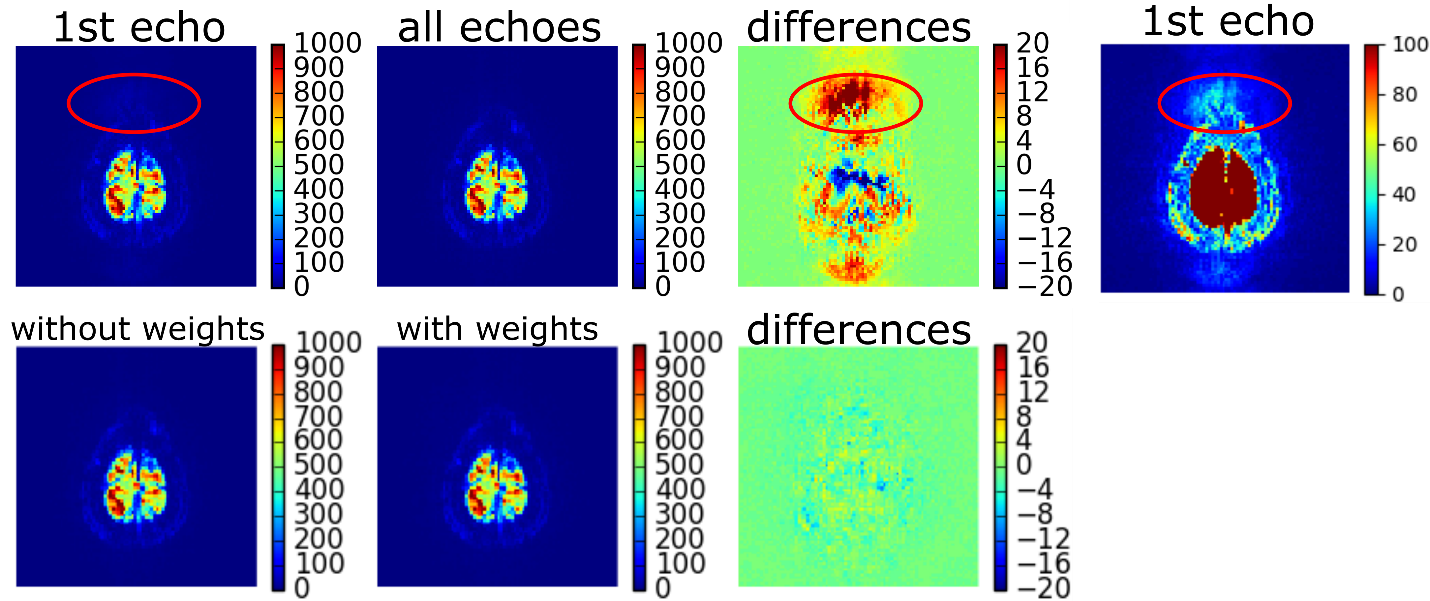


**Figure S1**: A comparison of different multi-band multi-echo image reconstruction methods. These images demonstrate the reconstruction results of one slice of the second echo acquisition. The first row is the comparison between computing kernel matrix using only the first echo and using all the echoes. Note that neither of them weighted k-space signal during reconstruction. The red circle indicates the location of the artifact. The fourth image on the first row is the same as the first image but with a different color display range to emphasize the location of the artefact. The second row is the comparison between computing kernel matrix between with k-space weighting and without k-space weighting.


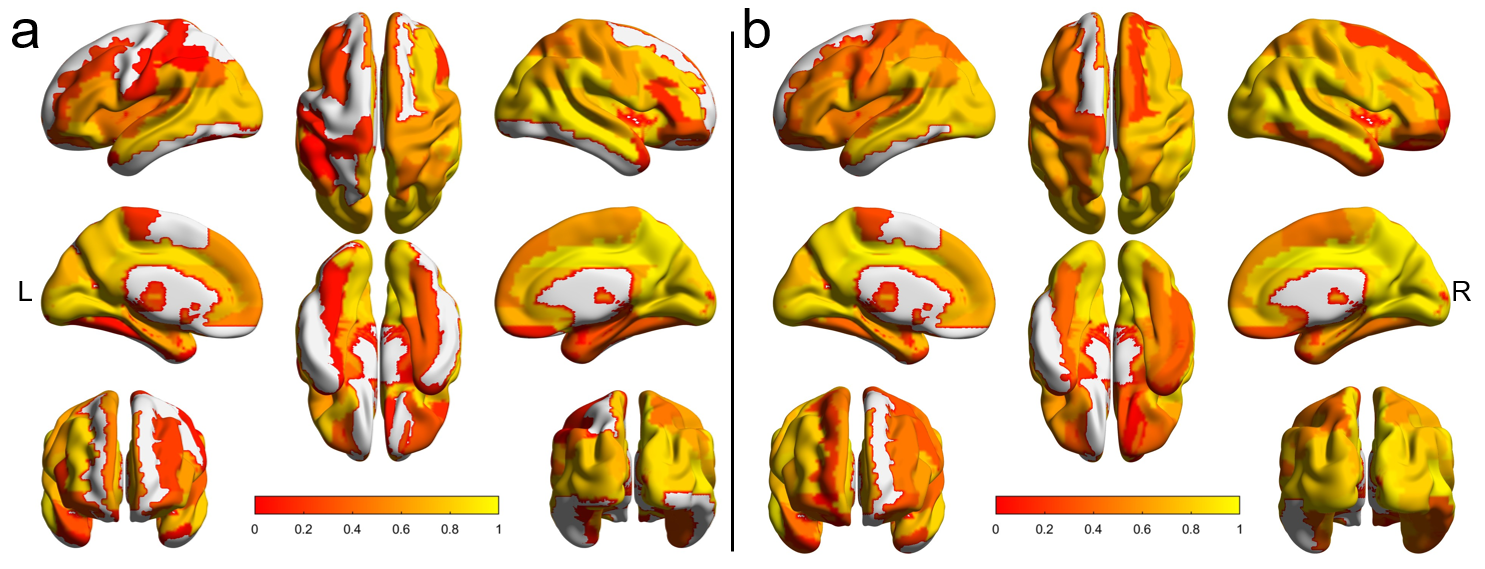


**Figure S2**: Surface renderings of intraclass correlation coefficients for baseline cerebral blood flow (CBF) calculated in regions of interest defined by the Automated Anatomical Labeling (AAL) atlas. Results are shown for the low-resolution M2-PCASL data: a) unsmoothed data; b) data smoothed with a 3-mm FWHM Gaussian kernel.


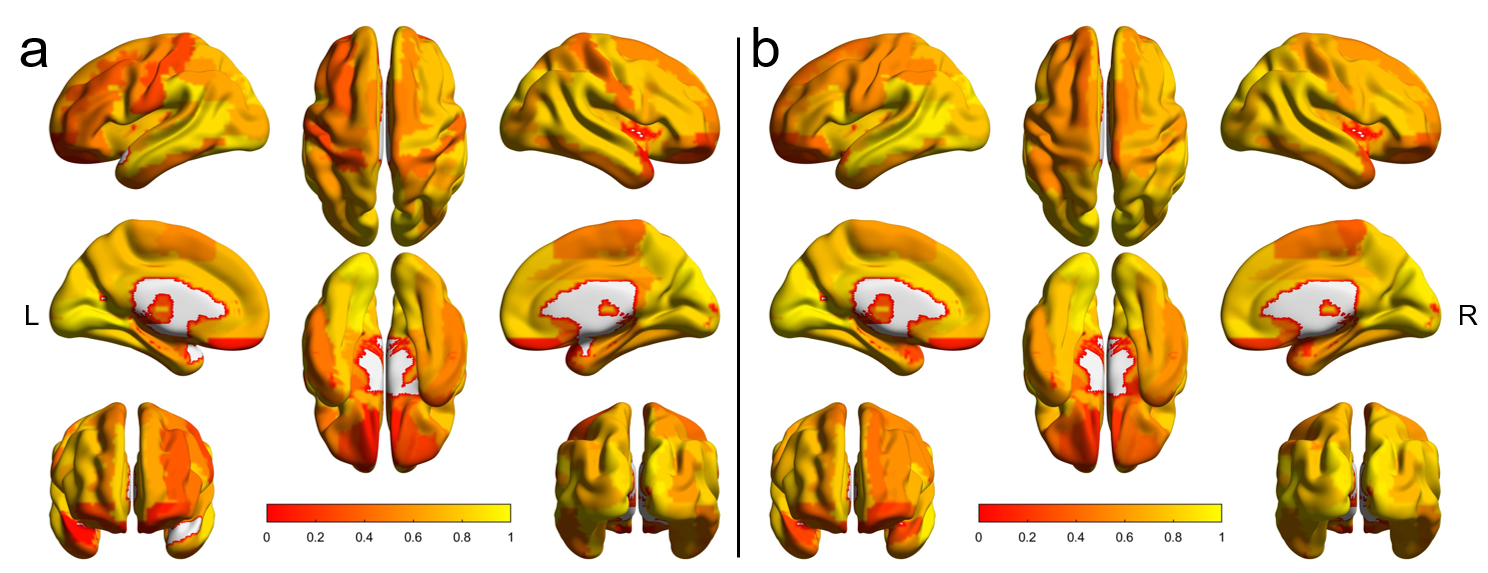


**Figure S3:** Surface renderings of intraclass correlation coefficients for baseline cerebral blood flow (CBF) calculated in regions of interest defined by the Automated Anatomical Labeling (AAL) atlas. Results are shown for the high-resolution M2-PCASL data: a) unsmoothed data; b) data smoothed with a 3-mm FWHM Gaussian kernel.


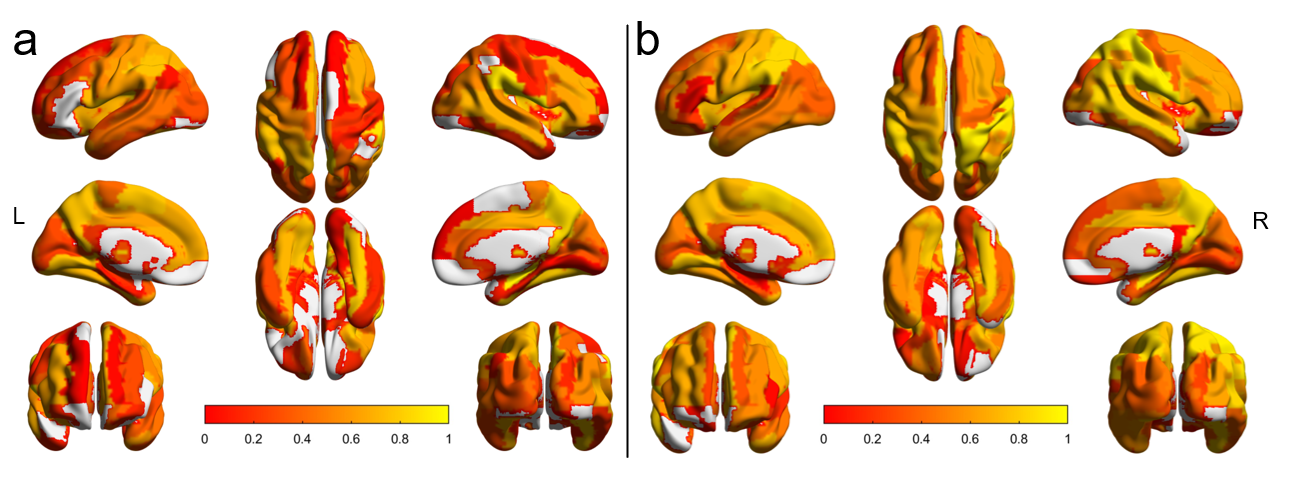


**Figure S4**: Surface renderings of intraclass correlation coefficients for blood oxygen level-dependent (BOLD) cerebrovascular reactivity (CVR) calculated in regions of interest defined by the Automated Anatomical Labeling (AAL) atlas: a) no BOLD smoothing; b) BOLD smoothing with a 3-mm FWHM Gaussian kernel.

**
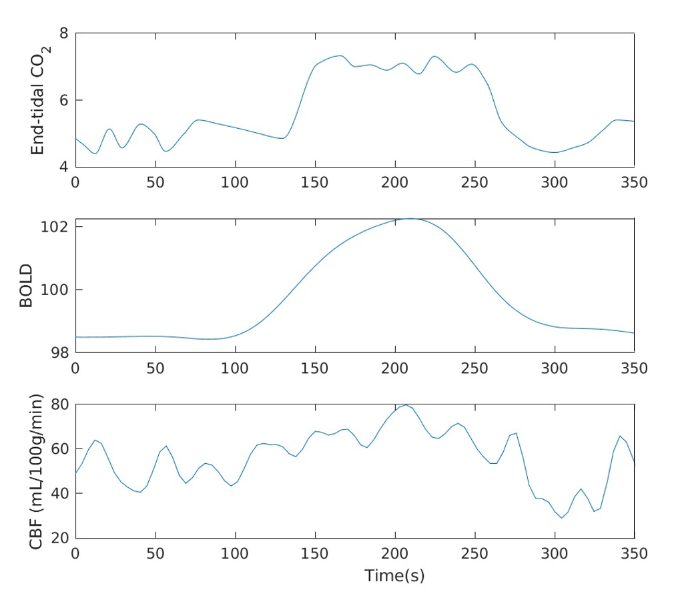
**

**Figure S5**: Average gray matter blood oxygen level-dependent (BOLD) and cerebral blood flow (CBF) signal with temporal smoothing during hypercapnia in a representative subject. In this figure, BOLD data is not spatially smoothed while the control-label pairs from the arterial spin labeled data is smoothed with a 5-mm FWHM Gaussian kernel.

**Tables**

| **Baseline CBF ICC (95% CI) in AAL ROIs from Low-resolution M2-PCASL Data** | | | |
| --- | --- | --- | --- |
| **ROI** | **No Smoothing** | **FWHM = 3 mm** | **FWHM = 5 mm** |
| Amygdala_L | 0.43 (-0.22<ICC<0.83) | 0.23 (-0.28<ICC<0.72) | 0.37 (-0.23<ICC<0.80) |
| Amygdala_R | 0.03 (-0.70<ICC<0.66) | 0.23 (-0.46<ICC<0.75) | 0.19 (-0.47<ICC<0.73) |
| Angular_L | 0.43 (-0.32<ICC<0.84) | 0.56 (-0.07<ICC<0.88) | 0.66 (0.09<ICC<0.91) |
| Angular_R | 0.66 (0.11<ICC<0.91) | 0.76 (0.29<ICC<0.94) | 0.78 (0.31<ICC<0.95) |
| Calcarine_L | 0.87 (0.55<ICC<0.97) | 0.87 (0.52<ICC<0.97) | 0.90 (0.62<ICC<0.98) |
| Calcarine_R | 0.87 (0.56<ICC<0.97) | 0.92 (0.72<ICC<0.98) | 0.93 (0.75<ICC<0.98) |
| Caudate_L | 0.49 (-0.26<ICC<0.86) | 0.53 (-0.18<ICC<0.87) | 0.38 (-0.36<ICC<0.82) |
| Caudate_R | 0.24 (-0.57<ICC<0.77) | 0.33 (-0.46<ICC<0.80) | 0.33 (-0.46<ICC<0.80) |
| Cingulum_Ant_L | 0.74 (0.19<ICC<0.94) | 0.76 (0.22<ICC<0.94) | 0.75 (0.23<ICC<0.94) |
| Cingulum_Ant_R | 0.77 (0.26<ICC<0.94) | 0.84 (0.44<ICC<0.96) | 0.86 (0.48<ICC<0.97) |
| Cingulum_Mid_L | 0.87 (0.56<ICC<0.97) | 0.96 (0.83<ICC<0.99) | 0.95 (0.79<ICC<0.99) |
| Cingulum_Mid_R | 0.95 (0.81<ICC<0.99) | 0.97 (0.88<ICC<0.99) | 0.95 (0.82<ICC<0.99) |
| Cingulum_Post_L | 0.82 (0.40<ICC<0.96) | 0.92 (0.69<ICC<0.98) | 0.90 (0.65<ICC<0.98) |
| Cingulum_Post_R | 0.66 (0.01<ICC<0.91) | 0.85 (0.50<ICC<0.96) | 0.91 (0.69<ICC<0.98) |
| Cuneus_L | 0.70 (0.17<ICC<0.92) | 0.72 (0.16<ICC<0.93) | 0.78 (0.28<ICC<0.94) |
| Cuneus_R | 0.86 (0.52<ICC<0.96) | 0.91 (0.68<ICC<0.98) | 0.91 (0.66<ICC<0.98) |
| Frontal_Inf_Oper_L | 0.66 (0.11<ICC<0.91) | 0.78 (0.33<ICC<0.95) | 0.82 (0.42<ICC<0.95) |
| Frontal_Inf_Oper_R | 0.76 (0.23<ICC<0.94) | 0.89 (0.59<ICC<0.97) | 0.92 (0.71<ICC<0.98) |
| Frontal_Inf_Orb_L | 0.81 (0.34<ICC<0.95) | 0.60 (0.02<ICC<0.89) | 0.65 (0.08<ICC<0.91) |
| Frontal_Inf_Orb_R | 0.33 (-0.39<ICC<0.80) | 0.48 (-0.23<ICC<0.86) | 0.53 (-0.18<ICC<0.87) |
| Frontal_Inf_Tri_L | 0.61 (0.03<ICC<0.89) | 0.61 (0.04<ICC<0.89) | 0.64 (0.08<ICC<0.90) |
| Frontal_Inf_Tri_R | 0.17 (-0.60<ICC<0.73) | 0.66 (0.02<ICC<0.91) | 0.75 (0.20<ICC<0.94) |
| Frontal_Med_Orb_L | 0.52 (-0.10<ICC<0.86) | 0.68 (0.13<ICC<0.92) | 0.50 (-0.13<ICC<0.86) |
| Frontal_Med_Orb_R | 0.68 (0.05<ICC<0.92) | 0.79 (0.35<ICC<0.95) | 0.81 (0.40<ICC<0.95) |
| Frontal_Mid_L | 0.24 (-0.54<ICC<0.77) | 0.47 (-0.29<ICC<0.86) | 0.47 (-0.31<ICC<0.85) |
| Frontal_Mid_Orb_L | 0.36 (-0.45<ICC<0.81) | 0.34 (-0.39<ICC<0.80) | 0.43 (-0.25<ICC<0.83) |
| Frontal_Mid_Orb_R | 0.34 (-0.33<ICC<0.80) | 0.41 (-0.28<ICC<0.82) | 0.50 (-0.19<ICC<0.86) |
| Frontal_Mid_R | 0.84 (0.43<ICC<0.96) | 0.80 (0.33<ICC<0.95) | 0.81 (0.35<ICC<0.95) |
| Frontal_Sup_L | -0.28 (-0.94<ICC<0.49) | -0.11 (-0.83<ICC<0.60) | 0.02 (-0.75<ICC<0.67) |
| Frontal_Sup_Medial_L | 0.60 (-0.08<ICC<0.89) | 0.70 (0.15<ICC<0.92) | 0.75 (0.24<ICC<0.94) |
| Frontal_Sup_Medial_R | 0.51 (-0.24<ICC<0.87) | 0.61 (-0.08<ICC<0.90) | 0.70 (0.10<ICC<0.92) |
| Frontal_Sup_Orb_L | -0.24 (-0.85<ICC<0.50) | -0.07 (-0.76<ICC<0.61) | -0.12 (-0.74<ICC<0.56) |
| Frontal_Sup_Orb_R | -0.18 (-0.89<ICC<0.55) | 0.02 (-0.76<ICC<0.67) | 0.08 (-0.70<ICC<0.70) |
| Frontal_Sup_R | -0.06 (-0.72<ICC<0.61) | 0.21 (-0.57<ICC<0.75) | 0.27 (-0.53<ICC<0.78) |
| Fusiform_L | 0.06 (-0.63<ICC<0.68) | 0.40 (-0.31<ICC<0.83) | 0.52 (-0.14<ICC<0.86) |
| Fusiform_R | 0.36 (-0.39<ICC<0.81) | 0.49 (-0.19<ICC<0.85) | 0.49 (-0.22<ICC<0.86) |
| GM | 0.88 (0.58<ICC<0.97) | 0.89 (0.62<ICC<0.97) | 0.90 (0.66<ICC<0.98) |
| Heschl_L | 0.39 (-0.21<ICC<0.81) | 0.53 (-0.14<ICC<0.87) | 0.53 (-0.20<ICC<0.87) |
| Heschl_R | 0.78 (0.26<ICC<0.95) | 0.65 (-0.01<ICC<0.91) | 0.67 (0.01<ICC<0.91) |
| Hippocampus_L | 0.62 (0.06<ICC<0.90) | 0.65 (0.07<ICC<0.91) | 0.64 (0.07<ICC<0.90) |
| Hippocampus_R | 0.30 (-0.37<ICC<0.78) | 0.66 (0.12<ICC<0.91) | 0.60 (0.02<ICC<0.89) |
| Insula_L | 0.51 (-0.18<ICC<0.86) | 0.51 (-0.17<ICC<0.86) | 0.50 (-0.21<ICC<0.86) |
| Insula_R | 0.87 (0.54<ICC<0.97) | 0.83 (0.40<ICC<0.96) | 0.83 (0.41<ICC<0.96) |
| Lingual_L | 0.87 (0.54<ICC<0.97) | 0.91 (0.65<ICC<0.98) | 0.92 (0.68<ICC<0.98) |
| Lingual_R | 0.87 (0.55<ICC<0.97) | 0.94 (0.77<ICC<0.99) | 0.95 (0.80<ICC<0.99) |
| Occipital_Inf_L | NA | 0.90 (0.61<ICC<0.98) | 0.90 (0.63<ICC<0.98) |
| Occipital_Inf_R | NA | 0.78 (0.34<ICC<0.95) | 0.78 (0.33<ICC<0.94) |
| Occipital_Mid_L | 0.80 (0.33<ICC<0.95) | 0.81 (0.37<ICC<0.95) | 0.81 (0.36<ICC<0.95) |
| Occipital_Mid_R | 0.82 (0.39<ICC<0.95) | 0.82 (0.37<ICC<0.96) | 0.87 (0.53<ICC<0.97) |
| Occipital_Sup_L | 0.73 (0.16<ICC<0.93) | 0.82 (0.38<ICC<0.96) | 0.83 (0.43<ICC<0.96) |
| Occipital_Sup_R | 0.87 (0.54<ICC<0.97) | 0.87 (0.51<ICC<0.97) | 0.88 (0.56<ICC<0.97) |
| Olfactory_L | -0.09 (-0.79<ICC<0.60) | -0.22 (-0.86<ICC<0.52) | -0.12 (-0.82<ICC<0.58) |
| Olfactory_R | 0.83 (0.40<ICC<0.96) | 0.40 (-0.33<ICC<0.82) | 0.39 (-0.32<ICC<0.82) |
| Pallidum_L | 0.22 (-0.44<ICC<0.74) | 0.35 (-0.29<ICC<0.80) | 0.41 (-0.28<ICC<0.82) |
| Pallidum_R | 0.60 (-0.10<ICC<0.90) | 0.73 (0.14<ICC<0.93) | 0.73 (0.18<ICC<0.93) |
| Paracentral_Lobule_L | 0.19 (-0.60<ICC<0.74) | 0.29 (-0.50<ICC<0.79) | 0.50 (-0.24<ICC<0.86) |
| Paracentral_Lobule_R | 0.61 (0.03<ICC<0.89) | 0.69 (0.16<ICC<0.92) | 0.67 (0.11<ICC<0.91) |
| ParaHippocampal_L | 0.63 (-0.01<ICC<0.90) | 0.69 (0.11<ICC<0.92) | 0.71 (0.19<ICC<0.93) |
| ParaHippocampal_R | 0.34 (-0.35<ICC<0.80) | 0.65 (0.03<ICC<0.91) | 0.64 (0.02<ICC<0.90) |
| Parietal_Inf_L | 0.03 (-0.54<ICC<0.63) | 0.69 (0.13<ICC<0.92) | 0.73 (0.20<ICC<0.93) |
| Parietal_Inf_R | 0.59 (-0.08<ICC<0.89) | 0.81 (0.41<ICC<0.95) | 0.86 (0.54<ICC<0.97) |
| Parietal_Sup_L | -0.09 (-0.83<ICC<0.61) | 0.43 (-0.20<ICC<0.83) | 0.61 (0.03<ICC<0.89) |
| Parietal_Sup_R | 0.46 (-0.11<ICC<0.84) | 0.60 (0.01<ICC<0.89) | 0.67 (0.01<ICC<0.92) |
| Postcentral_L | 0.10 (-0.63<ICC<0.70) | 0.49 (-0.27<ICC<0.86) | 0.61 (-0.10<ICC<0.90) |
| Postcentral_R | 0.64 (0.04<ICC<0.90) | 0.69 (0.13<ICC<0.92) | 0.74 (0.20<ICC<0.93) |
| Precentral_L | -0.08 (-0.82<ICC<0.62) | 0.33 (-0.47<ICC<0.80) | 0.42 (-0.37<ICC<0.84) |
| Precentral_R | 0.63 (-0.04<ICC<0.90) | 0.81 (0.35<ICC<0.95) | 0.84 (0.41<ICC<0.96) |
| Precuneus_L | 0.79 (0.35<ICC<0.95) | 0.88 (0.57<ICC<0.97) | 0.88 (0.56<ICC<0.97) |
| Precuneus_R | 0.95 (0.78<ICC<0.99) | 0.94 (0.77<ICC<0.99) | 0.95 (0.79<ICC<0.99) |
| Putamen_L | 0.19 (-0.45<ICC<0.73) | 0.19 (-0.45<ICC<0.72) | 0.19 (-0.44<ICC<0.73) |
| Putamen_R | 0.47 (-0.26<ICC<0.85) | 0.60 (-0.02<ICC<0.89) | 0.62 (0.02<ICC<0.90) |
| Rectus_L | -0.21 (-0.69<ICC<0.46) | -0.02 (-0.50<ICC<0.58) | -0.13 (-0.73<ICC<0.55) |
| Rectus_R | 0.10 (-0.70<ICC<0.71) | 0.30 (-0.48<ICC<0.79) | 0.41 (-0.32<ICC<0.83) |
| Rolandic_Oper_L | 0.66 (0.11<ICC<0.91) | 0.71 (0.20<ICC<0.93) | 0.72 (0.21<ICC<0.93) |
| Rolandic_Oper_R | 0.87 (0.55<ICC<0.97) | 0.86 (0.52<ICC<0.97) | 0.84 (0.46<ICC<0.96) |
| Supp_Motor_Area_L | -0.12 (-0.67<ICC<0.55) | -0.10 (-0.71<ICC<0.58) | 0.10 (-0.62<ICC<0.69) |
| Supp_Motor_Area_R | 0.63 (0.00<ICC<0.90) | 0.48 (-0.29<ICC<0.86) | 0.50 (-0.26<ICC<0.86) |
| SupraMarginal_L | 0.70 (0.16<ICC<0.92) | 0.83 (0.45<ICC<0.96) | 0.83 (0.44<ICC<0.96) |
| SupraMarginal_R | 0.89 (0.59<ICC<0.97) | 0.91 (0.68<ICC<0.98) | 0.90 (0.65<ICC<0.98) |
| Temporal_Inf_L | -0.38 (-0.88<ICC<0.36) | -0.20 (-0.82<ICC<0.52) | 0.12 (-0.63<ICC<0.71) |
| Temporal_Inf_R | -0.06 (-0.73<ICC<0.61) | 0.37 (-0.44<ICC<0.82) | 0.59 (-0.12<ICC<0.89) |
| Temporal_Mid_L | 0.81 (0.40<ICC<0.95) | 0.84 (0.48<ICC<0.96) | 0.86 (0.52<ICC<0.97) |
| Temporal_Mid_R | 0.92 (0.71<ICC<0.98) | 0.95 (0.81<ICC<0.99) | 0.96 (0.83<ICC<0.99) |
| Temporal_Pole_Mid_L | 0.03 (-0.40<ICC<0.59) | 0.23 (-0.40<ICC<0.74) | 0.20 (-0.48<ICC<0.73) |
| Temporal_Pole_Mid_R | 0.09 (-0.70<ICC<0.70) | 0.12 (-0.64<ICC<0.71) | 0.24 (-0.52<ICC<0.76) |
| Temporal_Pole_Sup_L | 0.72 (0.20<ICC<0.93) | 0.73 (0.17<ICC<0.93) | 0.74 (0.21<ICC<0.94) |
| Temporal_Pole_Sup_R | 0.31 (-0.28<ICC<0.77) | 0.43 (-0.17<ICC<0.83) | 0.48 (-0.12<ICC<0.85) |
| Temporal_Sup_L | 0.38 (-0.42<ICC<0.82) | 0.40 (-0.39<ICC<0.83) | 0.44 (-0.34<ICC<0.85) |
| Temporal_Sup_R | 0.72 (0.14<ICC<0.93) | 0.74 (0.18<ICC<0.94) | 0.77 (0.25<ICC<0.94) |
| Thalamus_L | 0.60 (0.01<ICC<0.89) | 0.70 (0.16<ICC<0.92) | 0.68 (0.12<ICC<0.91) |
| Thalamus_R | 0.57 (-0.15<ICC<0.89) | 0.64 (0.00<ICC<0.91) | 0.66 (0.05<ICC<0.91) |
| Whole Brain | 0.85 (0.49<ICC<0.96) | 0.89 (0.59<ICC<0.97) | 0.90 (0.63<ICC<0.98) |
| WM | 0.83 (0.40<ICC<0.96) | 0.87 (0.53<ICC<0.97) | 0.90 (0.61<ICC<0.98) |

**Table S1**: Intraclass correlation coefficients (ICCs) and 95% confidence intervals (CIs) computed from average baseline cerebral blood flow (CBF) estimates in the regions of interest (ROIs) provided by the Automated Anatomical Labeling (AAL) atlas. Results are shown for the low-resolution M2-PCASL data with no smoothing and with smoothing with 3 and 5-mm FWHM Gaussian kernels. Abbreviations: L: left, R: right, Ant: Anterior, Mid: middle, Post: posterior, Oper: operculum, Orb: orbital, Tri: triangularis, Sup: superior, GM: gray matter, WM: white matter.

| **Baseline CBF ICC (95% CI) in AAL ROIs from High-resolution M2-PCASL Data** | | | |
| --- | --- | --- | --- |
| **ROI** | **No Smoothing** | **FWHM = 3 mm** | **FWHM = 5 mm** |
| Amygdala_L | 0.41 (-0.20<ICC<0.82) | 0.44 (-0.21<ICC<0.83) | 0.53 (-0.11<ICC<0.87) |
| Amygdala_R | -0.09 (-0.71<ICC<0.58) | 0.16 (-0.54<ICC<0.72) | 0.16 (-0.57<ICC<0.73) |
| Angular_L | 0.65 (0.04<ICC<0.91) | 0.79 (0.36<ICC<0.95) | 0.84 (0.45<ICC<0.96) |
| Angular_R | 0.78 (0.29<ICC<0.95) | 0.84 (0.44<ICC<0.96) | 0.81 (0.39<ICC<0.95) |
| Calcarine_L | 0.93 (0.66<ICC<0.98) | 0.95 (0.54<ICC<0.99) | 0.95 (0.63<ICC<0.99) |
| Calcarine_R | 0.85 (0.46<ICC<0.96) | 0.87 (0.52<ICC<0.97) | 0.87 (0.52<ICC<0.97) |
| Caudate_L | 0.64 (0.02<ICC<0.91) | 0.58 (-0.11<ICC<0.89) | 0.55 (-0.17<ICC<0.88) |
| Caudate_R | 0.84 (0.42<ICC<0.96) | 0.83 (0.46<ICC<0.96) | 0.87 (0.54<ICC<0.97) |
| Cingulum_Ant_L | 0.77 (0.32<ICC<0.94) | 0.85 (0.51<ICC<0.96) | 0.87 (0.56<ICC<0.97) |
| Cingulum_Ant_R | 0.79 (0.33<ICC<0.95) | 0.82 (0.40<ICC<0.96) | 0.78 (0.30<ICC<0.95) |
| Cingulum_Mid_L | 0.76 (0.24<ICC<0.94) | 0.83 (0.42<ICC<0.96) | 0.80 (0.34<ICC<0.95) |
| Cingulum_Mid_R | 0.65 (0.08<ICC<0.91) | 0.70 (0.13<ICC<0.92) | 0.76 (0.27<ICC<0.94) |
| Cingulum_Post_L | 0.82 (0.43<ICC<0.96) | 0.88 (0.60<ICC<0.97) | 0.86 (0.52<ICC<0.97) |
| Cingulum_Post_R | 0.55 (-0.14<ICC<0.88) | 0.79 (0.35<ICC<0.95) | 0.82 (0.40<ICC<0.96) |
| Cuneus_L | 0.82 (0.39<ICC<0.96) | 0.89 (0.61<ICC<0.97) | 0.90 (0.61<ICC<0.98) |
| Cuneus_R | 0.93 (0.75<ICC<0.98) | 0.93 (0.72<ICC<0.98) | 0.94 (0.78<ICC<0.99) |
| Frontal_Inf_Oper_L | 0.80 (0.31<ICC<0.95) | 0.87 (0.52<ICC<0.97) | 0.88 (0.54<ICC<0.97) |
| Frontal_Inf_Oper_R | 0.67 (0.08<ICC<0.92) | 0.73 (0.21<ICC<0.93) | 0.76 (0.25<ICC<0.94) |
| Frontal_Inf_Orb_L | 0.50 (-0.26<ICC<0.86) | 0.43 (-0.36<ICC<0.84) | 0.47 (-0.31<ICC<0.85) |
| Frontal_Inf_Orb_R | 0.57 (-0.16<ICC<0.88) | 0.55 (-0.18<ICC<0.88) | 0.54 (-0.20<ICC<0.88) |
| Frontal_Inf_Tri_L | 0.46 (-0.25<ICC<0.85) | 0.70 (0.10<ICC<0.93) | 0.74 (0.17<ICC<0.93) |
| Frontal_Inf_Tri_R | 0.79 (0.32<ICC<0.95) | 0.82 (0.40<ICC<0.96) | 0.85 (0.46<ICC<0.96) |
| Frontal_Med_Orb_L | 0.70 (0.10<ICC<0.92) | 0.74 (0.18<ICC<0.94) | 0.74 (0.19<ICC<0.94) |
| Frontal_Med_Orb_R | 0.83 (0.42<ICC<0.96) | 0.86 (0.50<ICC<0.97) | 0.89 (0.60<ICC<0.97) |
| Frontal_Mid_L | 0.35 (-0.25<ICC<0.79) | 0.57 (-0.04<ICC<0.88) | 0.63 (0.04<ICC<0.90) |
| Frontal_Mid_Orb_L | 0.25 (-0.47<ICC<0.76) | 0.28 (-0.43<ICC<0.78) | 0.28 (-0.48<ICC<0.78) |
| Frontal_Mid_Orb_R | 0.24 (-0.55<ICC<0.77) | 0.37 (-0.35<ICC<0.82) | 0.46 (-0.31<ICC<0.85) |
| Frontal_Mid_R | 0.76 (0.23<ICC<0.94) | 0.75 (0.22<ICC<0.94) | 0.76 (0.24<ICC<0.94) |
| Frontal_Sup_L | 0.64 (0.08<ICC<0.90) | 0.44 (-0.18<ICC<0.83) | 0.54 (-0.07<ICC<0.87) |
| Frontal_Sup_Medial_L | 0.72 (0.20<ICC<0.93) | 0.74 (0.24<ICC<0.94) | 0.78 (0.32<ICC<0.95) |
| Frontal_Sup_Medial_R | 0.81 (0.40<ICC<0.95) | 0.80 (0.33<ICC<0.95) | 0.83 (0.42<ICC<0.96) |
| Frontal_Sup_Orb_L | 0.11 (-0.62<ICC<0.70) | 0.21 (-0.52<ICC<0.75) | 0.28 (-0.48<ICC<0.78) |
| Frontal_Sup_Orb_R | 0.31 (-0.33<ICC<0.78) | 0.43 (-0.18<ICC<0.83) | 0.50 (-0.13<ICC<0.86) |
| Frontal_Sup_R | 0.55 (-0.06<ICC<0.87) | 0.59 (-0.02<ICC<0.89) | 0.66 (0.09<ICC<0.91) |
| Fusiform_L | 0.81 (0.38<ICC<0.95) | 0.77 (0.25<ICC<0.94) | 0.78 (0.27<ICC<0.95) |
| Fusiform_R | 0.65 (0.05<ICC<0.91) | 0.68 (0.09<ICC<0.92) | 0.67 (0.06<ICC<0.91) |
| GM | 0.88 (0.56<ICC<0.97) | 0.86 (0.50<ICC<0.97) | 0.88 (0.57<ICC<0.97) |
| Heschl_L | 0.36 (-0.42<ICC<0.82) | 0.48 (-0.28<ICC<0.86) | 0.59 (-0.11<ICC<0.89) |
| Heschl_R | 0.72 (0.14<ICC<0.93) | 0.63 (-0.04<ICC<0.90) | 0.71 (0.13<ICC<0.93) |
| Hippocampus_L | 0.57 (-0.03<ICC<0.88) | 0.62 (0.05<ICC<0.90) | 0.67 (0.13<ICC<0.91) |
| Hippocampus_R | 0.63 (0.07<ICC<0.90) | 0.60 (0.02<ICC<0.89) | 0.58 (0.00<ICC<0.88) |
| Insula_L | 0.75 (0.27<ICC<0.94) | 0.80 (0.39<ICC<0.95) | 0.81 (0.38<ICC<0.95) |
| Insula_R | 0.77 (0.28<ICC<0.94) | 0.71 (0.14<ICC<0.93) | 0.75 (0.26<ICC<0.94) |
| Lingual_L | 0.95 (0.81<ICC<0.99) | 0.94 (0.75<ICC<0.99) | 0.94 (0.77<ICC<0.99) |
| Lingual_R | 0.84 (0.46<ICC<0.96) | 0.88 (0.58<ICC<0.97) | 0.85 (0.48<ICC<0.97) |
| Occipital_Inf_L | 0.91 (0.63<ICC<0.98) | 0.90 (0.60<ICC<0.98) | 0.93 (0.74<ICC<0.98) |
| Occipital_Inf_R | 0.73 (0.22<ICC<0.93) | 0.80 (0.34<ICC<0.95) | 0.84 (0.46<ICC<0.96) |
| Occipital_Mid_L | 0.68 (0.10<ICC<0.92) | 0.77 (0.27<ICC<0.94) | 0.79 (0.31<ICC<0.95) |
| Occipital_Mid_R | 0.61 (0.04<ICC<0.89) | 0.72 (0.16<ICC<0.93) | 0.73 (0.18<ICC<0.93) |
| Occipital_Sup_L | 0.87 (0.55<ICC<0.97) | 0.92 (0.70<ICC<0.98) | 0.94 (0.77<ICC<0.99) |
| Occipital_Sup_R | 0.84 (0.44<ICC<0.96) | 0.88 (0.55<ICC<0.97) | 0.90 (0.61<ICC<0.98) |
| Olfactory_L | 0.69 (0.13<ICC<0.92) | 0.68 (0.08<ICC<0.92) | 0.69 (0.09<ICC<0.92) |
| Olfactory_R | 0.43 (-0.36<ICC<0.84) | 0.45 (-0.31<ICC<0.85) | 0.48 (-0.29<ICC<0.86) |
| Pallidum_L | -0.09 (-0.79<ICC<0.60) | 0.12 (-0.66<ICC<0.71) | 0.18 (-0.61<ICC<0.74) |
| Pallidum_R | 0.40 (-0.39<ICC<0.83) | 0.44 (-0.34<ICC<0.84) | 0.39 (-0.39<ICC<0.83) |
| Paracentral_Lobule_L | 0.63 (-0.04<ICC<0.91) | 0.62 (-0.06<ICC<0.90) | 0.64 (0.01<ICC<0.91) |
| Paracentral_Lobule_R | 0.44 (-0.34<ICC<0.84) | 0.37 (-0.40<ICC<0.82) | 0.52 (-0.22<ICC<0.87) |
| ParaHippocampal_L | 0.63 (0.00<ICC<0.90) | 0.75 (0.21<ICC<0.94) | 0.73 (0.19<ICC<0.93) |
| ParaHippocampal_R | 0.57 (-0.03<ICC<0.88) | 0.61 (-0.02<ICC<0.90) | 0.69 (0.11<ICC<0.92) |
| Parietal_Inf_L | 0.59 (-0.05<ICC<0.89) | 0.52 (-0.21<ICC<0.87) | 0.52 (-0.21<ICC<0.87) |
| Parietal_Inf_R | 0.64 (0.02<ICC<0.90) | 0.74 (0.25<ICC<0.93) | 0.78 (0.33<ICC<0.94) |
| Parietal_Sup_L | 0.73 (0.23<ICC<0.93) | 0.72 (0.19<ICC<0.93) | 0.71 (0.16<ICC<0.93) |
| Parietal_Sup_R | 0.61 (0.04<ICC<0.89) | 0.81 (0.39<ICC<0.95) | 0.80 (0.37<ICC<0.95) |
| Postcentral_L | 0.28 (-0.43<ICC<0.77) | 0.55 (-0.15<ICC<0.88) | 0.56 (-0.13<ICC<0.88) |
| Postcentral_R | 0.44 (-0.14<ICC<0.83) | 0.61 (0.01<ICC<0.89) | 0.67 (0.10<ICC<0.91) |
| Precentral_L | 0.52 (-0.21<ICC<0.87) | 0.56 (-0.07<ICC<0.88) | 0.62 (0.01<ICC<0.90) |
| Precentral_R | 0.80 (0.36<ICC<0.95) | 0.72 (0.19<ICC<0.93) | 0.77 (0.29<ICC<0.94) |
| Precuneus_L | 0.75 (0.24<ICC<0.94) | 0.76 (0.24<ICC<0.94) | 0.75 (0.22<ICC<0.94) |
| Precuneus_R | 0.86 (0.48<ICC<0.97) | 0.83 (0.39<ICC<0.96) | 0.84 (0.42<ICC<0.96) |
| Putamen_L | 0.72 (0.18<ICC<0.93) | 0.74 (0.19<ICC<0.93) | 0.75 (0.23<ICC<0.94) |
| Putamen_R | 0.69 (0.07<ICC<0.92) | 0.71 (0.12<ICC<0.93) | 0.74 (0.16<ICC<0.93) |
| Rectus_L | 0.08 (-0.65<ICC<0.69) | 0.12 (-0.64<ICC<0.71) | 0.22 (-0.57<ICC<0.76) |
| Rectus_R | 0.11 (-0.60<ICC<0.70) | 0.21 (-0.53<ICC<0.75) | 0.30 (-0.48<ICC<0.79) |
| Rolandic_Oper_L | 0.85 (0.51<ICC<0.96) | 0.90 (0.65<ICC<0.98) | 0.94 (0.77<ICC<0.99) |
| Rolandic_Oper_R | 0.72 (0.16<ICC<0.93) | 0.77 (0.27<ICC<0.94) | 0.76 (0.23<ICC<0.94) |
| Supp_Motor_Area_L | 0.54 (-0.10<ICC<0.87) | 0.60 (-0.04<ICC<0.89) | 0.60 (-0.01<ICC<0.89) |
| Supp_Motor_Area_R | 0.51 (-0.25<ICC<0.87) | 0.49 (-0.25<ICC<0.86) | 0.55 (-0.15<ICC<0.88) |
| SupraMarginal_L | 0.90 (0.65<ICC<0.98) | 0.90 (0.66<ICC<0.98) | 0.91 (0.68<ICC<0.98) |
| SupraMarginal_R | 0.85 (0.43<ICC<0.97) | 0.85 (0.51<ICC<0.96) | 0.87 (0.52<ICC<0.97) |
| Temporal_Inf_L | 0.46 (-0.25<ICC<0.85) | 0.61 (-0.03<ICC<0.89) | 0.65 (-0.01<ICC<0.91) |
| Temporal_Inf_R | 0.50 (-0.21<ICC<0.86) | 0.57 (-0.16<ICC<0.89) | 0.56 (-0.18<ICC<0.88) |
| Temporal_Mid_L | 0.87 (0.51<ICC<0.97) | 0.92 (0.69<ICC<0.98) | 0.92 (0.69<ICC<0.98) |
| Temporal_Mid_R | 0.82 (0.39<ICC<0.96) | 0.81 (0.35<ICC<0.95) | 0.82 (0.38<ICC<0.96) |
| Temporal_Pole_Mid_L | 0.70 (0.10<ICC<0.92) | 0.62 (-0.04<ICC<0.90) | 0.49 (-0.25<ICC<0.86) |
| Temporal_Pole_Mid_R | 0.34 (-0.22<ICC<0.78) | 0.43 (-0.14<ICC<0.83) | 0.37 (-0.20<ICC<0.80) |
| Temporal_Pole_Sup_L | -0.08 (-0.82<ICC<0.62) | 0.30 (-0.51<ICC<0.79) | 0.40 (-0.38<ICC<0.83) |
| Temporal_Pole_Sup_R | 0.05 (-0.72<ICC<0.68) | 0.27 (-0.54<ICC<0.78) | 0.37 (-0.43<ICC<0.82) |
| Temporal_Sup_L | 0.65 (-0.02<ICC<0.91) | 0.72 (0.12<ICC<0.93) | 0.73 (0.16<ICC<0.93) |
| Temporal_Sup_R | 0.82 (0.41<ICC<0.96) | 0.82 (0.38<ICC<0.96) | 0.85 (0.47<ICC<0.96) |
| Thalamus_L | 0.76 (0.24<ICC<0.94) | 0.80 (0.32<ICC<0.95) | 0.80 (0.31<ICC<0.95) |
| Thalamus_R | 0.75 (0.22<ICC<0.94) | 0.79 (0.32<ICC<0.95) | 0.83 (0.42<ICC<0.96) |
| Whole Brain | 0.90 (0.63<ICC<0.98) | 0.90 (0.61<ICC<0.98) | 0.90 (0.63<ICC<0.98) |
| WM | 0.88 (0.56<ICC<0.97) | 0.93 (0.74<ICC<0.98) | 0.92 (0.70<ICC<0.98) |

**Table S2**: Intraclass correlation coefficients (ICCs) and 95% confidence intervals (CIs) computed from average baseline cerebral blood flow (CBF) estimates in the regions of interest (ROIs) provided by the Automated Anatomical Labeling (AAL) atlas. Results are shown for the high-resolution M2-PCASL data with no smoothing and smoothing with 3 and 5-mm FWHM Gaussian kernels. Abbreviations: L: left, R: right, Ant: Anterior, Mid: middle, Post: posterior, Oper: operculum, Orb: orbital, Tri: triangularis, Sup: superior, GM: gray matter, WM: white matter.

| **BOLD CVR ICC (95% CI) in AAL ROIs from Low-resolution M2-PCASL Data** | | | |  |
| --- | --- | --- | --- | --- |
| **ROI** | **No Smoothing** | **FWHM = 3 mm** | **FWHM = 5 mm** | |
| Amygdala_L | -0.09 (-0.66<ICC<0.57) | 0.01 (-0.60<ICC<0.64) | 0.04 (-0.61<ICC<0.65) | |
| Amygdala_R | 0.12 (-0.64<ICC<0.71) | 0.32 (-0.49<ICC<0.80) | 0.42 (-0.34<ICC<0.84) | |
| Angular_L | 0.08 (-0.71<ICC<0.70) | 0.38 (-0.42<ICC<0.82) | 0.46 (-0.30<ICC<0.85) | |
| Angular_R | 0.54 (-0.16<ICC<0.88) | 0.83 (0.41<ICC<0.96) | 0.85 (0.46<ICC<0.96) | |
| Calcarine_L | 0.25 (-0.53<ICC<0.77) | 0.32 (-0.48<ICC<0.80) | 0.35 (-0.45<ICC<0.81) | |
| Calcarine_R | 0.34 (-0.47<ICC<0.81) | 0.35 (-0.41<ICC<0.81) | 0.35 (-0.41<ICC<0.81) | |
| Caudate_L | 0.32 (-0.46<ICC<0.80) | 0.58 (-0.12<ICC<0.89) | 0.66 (0.01<ICC<0.91) | |
| Caudate_R | 0.43 (-0.34<ICC<0.84) | 0.64 (-0.03<ICC<0.91) | 0.73 (0.15<ICC<0.93) | |
| Cingulum_Ant_L | 0.69 (0.07<ICC<0.92) | 0.72 (0.14<ICC<0.93) | 0.74 (0.20<ICC<0.94) | |
| Cingulum_Ant_R | 0.39 (-0.15<ICC<0.80) | 0.43 (-0.22<ICC<0.83) | 0.47 (-0.22<ICC<0.85) | |
| Cingulum_Mid_L | 0.62 (-0.06<ICC<0.90) | 0.69 (0.06<ICC<0.92) | 0.71 (0.10<ICC<0.93) | |
| Cingulum_Mid_R | 0.69 (0.16<ICC<0.92) | 0.75 (0.28<ICC<0.94) | 0.77 (0.29<ICC<0.94) | |
| Cingulum_Post_L | 0.22 (-0.28<ICC<0.71) | 0.34 (-0.26<ICC<0.79) | 0.42 (-0.22<ICC<0.83) | |
| Cingulum_Post_R | -0.31 (-0.78<ICC<0.40) | 0.05 (-0.52<ICC<0.65) | 0.22 (-0.40<ICC<0.73) | |
| Cuneus_L | 0.29 (-0.40<ICC<0.78) | 0.50 (-0.25<ICC<0.87) | 0.53 (-0.22<ICC<0.87) | |
| Cuneus_R | 0.48 (-0.22<ICC<0.85) | 0.46 (-0.26<ICC<0.85) | 0.48 (-0.24<ICC<0.86) | |
| Frontal_Inf_Oper_L | 0.73 (0.15<ICC<0.93) | 0.67 (0.02<ICC<0.92) | 0.65 (-0.02<ICC<0.91) | |
| Frontal_Inf_Oper_R | 0.38 (-0.29<ICC<0.82) | 0.71 (0.12<ICC<0.93) | 0.72 (0.14<ICC<0.93) | |
| Frontal_Inf_Orb_L | -0.01 (-0.60<ICC<0.62) | 0.22 (-0.42<ICC<0.74) | 0.29 (-0.37<ICC<0.77) | |
| Frontal_Inf_Orb_R | 0.64 (-0.02<ICC<0.91) | 0.65 (0.01<ICC<0.91) | 0.62 (-0.03<ICC<0.90) | |
| Frontal_Inf_Tri_L | -0.05 (-0.70<ICC<0.61) | 0.03 (-0.69<ICC<0.67) | 0.09 (-0.66<ICC<0.70) | |
| Frontal_Inf_Tri_R | 0.64 (0.07<ICC<0.90) | 0.51 (-0.11<ICC<0.86) | 0.46 (-0.18<ICC<0.84) | |
| Frontal_Med_Orb_L | -0.24 (-0.51<ICC<0.37) | -0.10 (-0.50<ICC<0.50) | 0.12 (-0.40<ICC<0.67) | |
| Frontal_Med_Orb_R | -0.19 (-0.78<ICC<0.52) | -0.41 (-0.95<ICC<0.36) | -0.48 (-0.99<ICC<0.30) | |
| Frontal_Mid_L | 0.30 (-0.29<ICC<0.77) | 0.54 (-0.10<ICC<0.87) | 0.54 (-0.09<ICC<0.87) | |
| Frontal_Mid_Orb_L | 0.16 (-0.50<ICC<0.71) | 0.56 (-0.11<ICC<0.88) | 0.66 (0.04<ICC<0.91) | |
| Frontal_Mid_Orb_R | 0.16 (-0.64<ICC<0.74) | -0.04 (-0.79<ICC<0.64) | -0.11 (-0.83<ICC<0.60) | |
| Frontal_Mid_R | 0.68 (0.11<ICC<0.92) | 0.68 (0.12<ICC<0.92) | 0.63 (0.02<ICC<0.90) | |
| Frontal_Sup_L | 0.04 (-0.74<ICC<0.68) | 0.33 (-0.38<ICC<0.80) | 0.37 (-0.36<ICC<0.82) | |
| Frontal_Sup_Medial_L | 0.68 (0.15<ICC<0.92) | 0.79 (0.34<ICC<0.95) | 0.82 (0.40<ICC<0.96) | |
| Frontal_Sup_Medial_R | 0.01 (-0.64<ICC<0.64) | 0.26 (-0.56<ICC<0.78) | 0.51 (-0.25<ICC<0.87) | |
| Frontal_Sup_Orb_L | 0.40 (-0.21<ICC<0.81) | 0.53 (-0.15<ICC<0.87) | 0.56 (-0.13<ICC<0.88) | |
| Frontal_Sup_Orb_R | -0.11 (-0.80<ICC<0.59) | 0.06 (-0.61<ICC<0.67) | 0.03 (-0.61<ICC<0.65) | |
| Frontal_Sup_R | 0.03 (-0.55<ICC<0.64) | 0.65 (0.06<ICC<0.91) | 0.67 (0.13<ICC<0.91) | |
| Fusiform_L | 0.71 (0.17<ICC<0.93) | 0.62 (0.05<ICC<0.90) | 0.56 (-0.03<ICC<0.88) | |
| Fusiform_R | 0.80 (0.35<ICC<0.95) | 0.81 (0.25<ICC<0.96) | 0.78 (0.17<ICC<0.95) | |
| GM | 0.57 (-0.12<ICC<0.89) | 0.86 (0.50<ICC<0.97) | 0.84 (0.43<ICC<0.96) | |
| Heschl_L | 0.55 (-0.16<ICC<0.88) | 0.47 (-0.31<ICC<0.85) | 0.46 (-0.31<ICC<0.85) | |
| Heschl_R | -0.29 (-0.89<ICC<0.47) | -0.10 (-0.84<ICC<0.60) | 0.14 (-0.66<ICC<0.73) | |
| Hippocampus_L | 0.19 (-0.31<ICC<0.70) | 0.07 (-0.39<ICC<0.63) | 0.09 (-0.38<ICC<0.63) | |
| Hippocampus_R | 0.93 (0.73<ICC<0.98) | 0.86 (0.51<ICC<0.97) | 0.82 (0.41<ICC<0.96) | |
| Insula_L | 0.70 (0.09<ICC<0.92) | 0.75 (0.19<ICC<0.94) | 0.77 (0.24<ICC<0.94) | |
| Insula_R | 0.79 (0.31<ICC<0.95) | 0.78 (0.31<ICC<0.94) | 0.76 (0.28<ICC<0.94) | |
| Lingual_L | 0.77 (0.25<ICC<0.94) | 0.79 (0.32<ICC<0.95) | 0.80 (0.37<ICC<0.95) | |
| Lingual_R | 0.04 (-0.61<ICC<0.66) | 0.23 (-0.49<ICC<0.75) | 0.31 (-0.42<ICC<0.79) | |
| Occipital_Mid_L | 0.32 (-0.49<ICC<0.80) | 0.37 (-0.43<ICC<0.82) | 0.37 (-0.43<ICC<0.82) | |
| Occipital_Mid_R | 0.54 (-0.10<ICC<0.87) | 0.64 (0.03<ICC<0.91) | 0.68 (0.07<ICC<0.92) | |
| Occipital_Sup_L | 0.44 (-0.32<ICC<0.84) | 0.46 (-0.32<ICC<0.85) | 0.43 (-0.35<ICC<0.84) | |
| Occipital_Sup_R | 0.16 (-0.45<ICC<0.70) | 0.26 (-0.48<ICC<0.77) | 0.32 (-0.44<ICC<0.79) | |
| Olfactory_L | -0.09 (-0.83<ICC<0.61) | 0.13 (-0.66<ICC<0.72) | 0.27 (-0.53<ICC<0.78) | |
| Olfactory_R | 0.22 (-0.52<ICC<0.75) | 0.46 (-0.22<ICC<0.84) | 0.58 (-0.02<ICC<0.89) | |
| Pallidum_L | 0.37 (-0.37<ICC<0.82) | 0.34 (-0.43<ICC<0.81) | 0.32 (-0.45<ICC<0.80) | |
| Pallidum_R | 0.58 (-0.14<ICC<0.89) | 0.62 (-0.06<ICC<0.90) | 0.65 (0.01<ICC<0.91) | |
| Paracentral_Lobule_L | 0.41 (-0.34<ICC<0.83) | 0.73 (0.14<ICC<0.93) | 0.78 (0.26<ICC<0.95) | |
| Paracentral_Lobule_R | 0.51 (-0.23<ICC<0.87) | 0.64 (-0.01<ICC<0.91) | 0.67 (0.06<ICC<0.92) | |
| ParaHippocampal_L | 0.10 (-0.63<ICC<0.70) | 0.32 (-0.48<ICC<0.80) | 0.40 (-0.36<ICC<0.83) | |
| ParaHippocampal_R | 0.11 (-0.68<ICC<0.71) | 0.37 (-0.44<ICC<0.82) | 0.49 (-0.27<ICC<0.86) | |
| Parietal_Inf_L | 0.77 (0.25<ICC<0.94) | 0.85 (0.47<ICC<0.97) | 0.87 (0.54<ICC<0.97) | |
| Parietal_Inf_R | -0.08 (-0.67<ICC<0.58) | 0.58 (0.00<ICC<0.88) | 0.72 (0.19<ICC<0.93) | |
| Parietal_Sup_L | 0.70 (0.10<ICC<0.93) | 0.86 (0.50<ICC<0.97) | 0.88 (0.59<ICC<0.97) | |
| Parietal_Sup_R | 0.42 (-0.32<ICC<0.83) | 0.90 (0.62<ICC<0.98) | 0.89 (0.59<ICC<0.97) | |
| Postcentral_L | 0.63 (0.01<ICC<0.90) | 0.72 (0.19<ICC<0.93) | 0.72 (0.18<ICC<0.93) | |
| Postcentral_R | 0.12 (-0.55<ICC<0.70) | 0.94 (0.78<ICC<0.99) | 0.94 (0.76<ICC<0.99) | |
| Precentral_L | 0.53 (-0.20<ICC<0.87) | 0.66 (0.01<ICC<0.91) | 0.65 (-0.01<ICC<0.91) | |
| Precentral_R | 0.21 (-0.57<ICC<0.75) | 0.40 (-0.40<ICC<0.83) | 0.45 (-0.34<ICC<0.85) | |
| Precuneus_L | 0.77 (0.31<ICC<0.94) | 0.76 (0.27<ICC<0.94) | 0.78 (0.31<ICC<0.94) | |
| Precuneus_R | 0.86 (0.53<ICC<0.97) | 0.88 (0.52<ICC<0.97) | 0.89 (0.57<ICC<0.97) | |
| Putamen_L | 0.66 (0.01<ICC<0.91) | 0.71 (0.11<ICC<0.93) | 0.73 (0.15<ICC<0.93) | |
| Putamen_R | 0.83 (0.43<ICC<0.96) | 0.83 (0.45<ICC<0.96) | 0.83 (0.46<ICC<0.96) | |
| Rectus_L | -0.53 (-1.06<ICC<0.28) | -0.21 (-0.90<ICC<0.54) | -0.09 (-0.82<ICC<0.61) | |
| Rectus_R | -0.07 (-0.75<ICC<0.60) | 0.11 (-0.57<ICC<0.70) | 0.29 (-0.42<ICC<0.78) | |
| Rolandic_Oper_L | 0.83 (0.44<ICC<0.96) | 0.84 (0.47<ICC<0.96) | 0.87 (0.54<ICC<0.97) | |
| Rolandic_Oper_R | 0.94 (0.77<ICC<0.99) | 0.93 (0.73<ICC<0.98) | 0.95 (0.78<ICC<0.99) | |
| Supp_Motor_Area_L | 0.78 (0.29<ICC<0.94) | 0.85 (0.47<ICC<0.97) | 0.84 (0.43<ICC<0.96) | |
| Supp_Motor_Area_R | -0.10 (-0.82<ICC<0.60) | 0.40 (-0.35<ICC<0.83) | 0.47 (-0.29<ICC<0.85) | |
| SupraMarginal_L | 0.62 (-0.04<ICC<0.90) | 0.84 (0.43<ICC<0.96) | 0.86 (0.50<ICC<0.97) | |
| SupraMarginal_R | 0.90 (0.55<ICC<0.98) | 0.91 (0.64<ICC<0.98) | 0.90 (0.63<ICC<0.98) | |
| Temporal_Inf_L | 0.29 (-0.45<ICC<0.78) | 0.57 (-0.01<ICC<0.88) | 0.57 (-0.01<ICC<0.88) | |
| Temporal_Inf_R | 0.18 (-0.57<ICC<0.73) | 0.41 (-0.30<ICC<0.83) | 0.40 (-0.27<ICC<0.82) | |
| Temporal_Mid_L | 0.45 (-0.31<ICC<0.85) | 0.48 (-0.26<ICC<0.86) | 0.52 (-0.19<ICC<0.87) | |
| Temporal_Mid_R | 0.70 (0.17<ICC<0.92) | 0.84 (0.46<ICC<0.96) | 0.84 (0.49<ICC<0.96) | |
| Temporal_Pole_Mid_L | 0.61 (-0.05<ICC<0.90) | 0.72 (0.17<ICC<0.93) | 0.77 (0.28<ICC<0.94) | |
| Temporal_Pole_Mid_R | 0.12 (-0.67<ICC<0.72) | -0.07 (-0.75<ICC<0.61) | -0.01 (-0.67<ICC<0.64) | |
| Temporal_Pole_Sup_L | 0.37 (-0.40<ICC<0.82) | 0.45 (-0.22<ICC<0.84) | 0.46 (-0.19<ICC<0.84) | |
| Temporal_Pole_Sup_R | -0.28 (-0.74<ICC<0.42) | -0.08 (-0.53<ICC<0.53) | 0.09 (-0.38<ICC<0.64) | |
| Temporal_Sup_L | 0.30 (-0.46<ICC<0.79) | 0.17 (-0.55<ICC<0.73) | 0.23 (-0.49<ICC<0.75) | |
| Temporal_Sup_R | 0.59 (-0.09<ICC<0.89) | 0.61 (-0.06<ICC<0.90) | 0.66 (0.03<ICC<0.91) | |
| Thalamus_L | 0.82 (0.37<ICC<0.96) | 0.88 (0.55<ICC<0.97) | 0.90 (0.63<ICC<0.98) | |
| Thalamus_R | 0.84 (0.12<ICC<0.97) | 0.91 (0.45<ICC<0.98) | 0.92 (0.64<ICC<0.98) | |
| Whole Brain | 0.65 (-0.03<ICC<0.91) | 0.84 (0.43<ICC<0.96) | 0.82 (0.37<ICC<0.96) | |
| WM | 0.51 (-0.09<ICC<0.86) | 0.82 (0.37<ICC<0.96) | 0.81 (0.33<ICC<0.95) | |

**Table S3**: Intraclass correlation coefficients (ICCs) and 95% confidence intervals (CIs) computed from average blood oxygen level-dependent (BOLD) cerebrovascular reactivity (CVR) estimates in the regions of interest (ROIs) provided by the Automated Anatomical Labeling (AAL) atlas. Results are shown for the low-resolution M2-PCASL data with no smoothing and smoothing with 3 and 5-mm FWHM Gaussian kernels. Abbreviations: L: left, R: right, Ant: Anterior, Mid: middle, Post: posterior, Oper: operculum, Orb: orbital, Tri: triangularis, Sup: superior, GM: gray matter, WM: white matter.

| **BOLD CVR and tCNR in 5% and 8% CO_2_ groups (Unsmoothed)** | | | | | | | | | | |
| --- | --- | --- | --- | --- | --- | --- | --- | --- | --- | --- |
|  | **BOLD tCNR** | | |  | | **BOLD CVR** | | | |  |
| **ROI** | **5% CO_2_** | **8% CO_2_** | **P-value** | |  | | **5% CO_2_** | **8% CO_2_** | **P-value** | |
| GM | 2.52±1.25 | 3.14±0.83 | 0.24 | |  | | 0.32 | 0.29 | 0.71 | |
| WM | 2.60±1.16 | 3.35±0.83 | 0.14 | |  | | 0.18 | 0.15 | 0.22 | |
| Whole Brain | 2.56±1.20 | 3.24±0.82 | 0.18 | |  | | 0.25 | 0.22 | 0.51 | |
|  |  |  |  | |  | |  |  |  | |

**Table S4**: Comparisons of blood oxygen level-dependent (BOLD) cerebrovascular reactivity (CVR) and temporal contrast to noise ratio (tCNR) between 5% and 8% inspired carbon dioxide (CO_2_) studies in gray matter (GM), white matter (WM), and whole brain regions of interest (ROIs). No BOLD smoothing.

| **BOLD CVR and tCNR in 5% and 8% CO_2_ groups (3-mm FWHM smoothed)** | | | | | | | | | | |
| --- | --- | --- | --- | --- | --- | --- | --- | --- | --- | --- |
|  | **BOLD tCNR** | | |  | | **BOLD CVR** | | | |  |
| **ROI** | **5% CO_2_** | **8% CO_2_** | **P-value** | |  | | **5% CO_2_** | **8% CO_2_** | **P-value** | |
| GM | 3.70±1.75 | 4.84±1.35 | 0.14 | |  | | 0.26 | 0.23 | 0.36 | |
| WM | 4.05±1.85 | 5.36±1.37 | 0.11 | |  | | 0.17 | 0.16 | 0.54 | |
| Whole Brain | 3.88±1.79 | 5.10±1.35 | 0.12 | |  | | 0.22 | 0.20 | 0.42 | |
|  |  |  |  | |  | |  |  |  | |

**Table S5**: Comparisons of blood oxygen level-dependent (BOLD) cerebrovascular reactivity (CVR) and temporal contrast to noise ratio (CNR) between 5% and 8% inspired carbon dioxide (CO_2_) studies in gray matter (GM), white matter (WM), and whole brain regions of interest (ROIs). BOLD data smoothed with a 3-mm FWHM Gaussian filter.

| **BOLD CVR and tCNR in 5% and 8% CO_2_ groups (5-mm FWHM smoothed)** | | | | | | | | | | |
| --- | --- | --- | --- | --- | --- | --- | --- | --- | --- | --- |
|  | **BOLD tCNR** | | |  | | **BOLD CVR** | | | |  |
| **ROI** | **5% CO_2_** | **8% CO_2_** | **P-value** | |  | | **5% CO_2_** | **8% CO_2_** | **P-value** | |
| GM | 4.25±1.97 | 5.76±1.72 | 0.10 | |  | | 0.25 | 0.22 | 0.37 | |
| WM | 4.66±2.15 | 6.37±1.68 | 0.08 | |  | | 0.17 | 0.16 | 0.52 | |
| Whole Brain | 4.46±2.05 | 6.07±1.69 | 0.09 | |  | | 0.21 | 0.19 | 0.42 | |

**Table S6**: Comparisons of blood oxygen level-dependent (BOLD) cerebrovascular reactivity (CVR) and temporal contrast to noise ratio (CNR) between 5% and 8% inspired carbon dioxide (CO_2_) studies in gray matter (GM), white matter (WM), and whole brain regions of interest (ROIs). BOLD data smoothed with a 5-mm FWHM Gaussian filter.


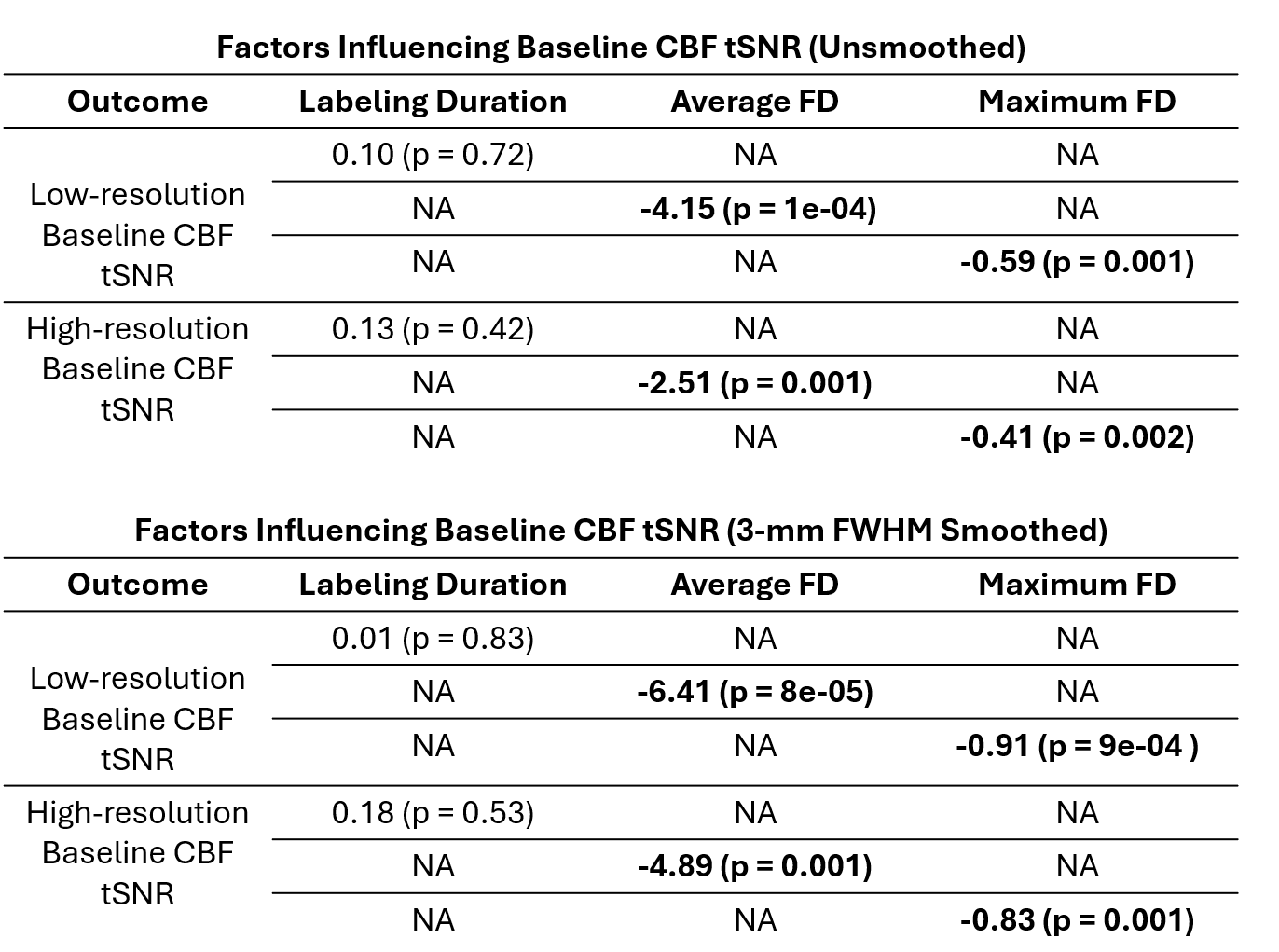


**Table S7**: Linear mixed effects models evaluating the factors influencing the temporal SNR (tSNR) of the low and high-resolution M2-PCASL baseline cerebral blood flow (CBF) time courses obtained from the unsmoothed M2-PCASL data. FD stands for framewise displacement. Bold font indicates statistical significance.


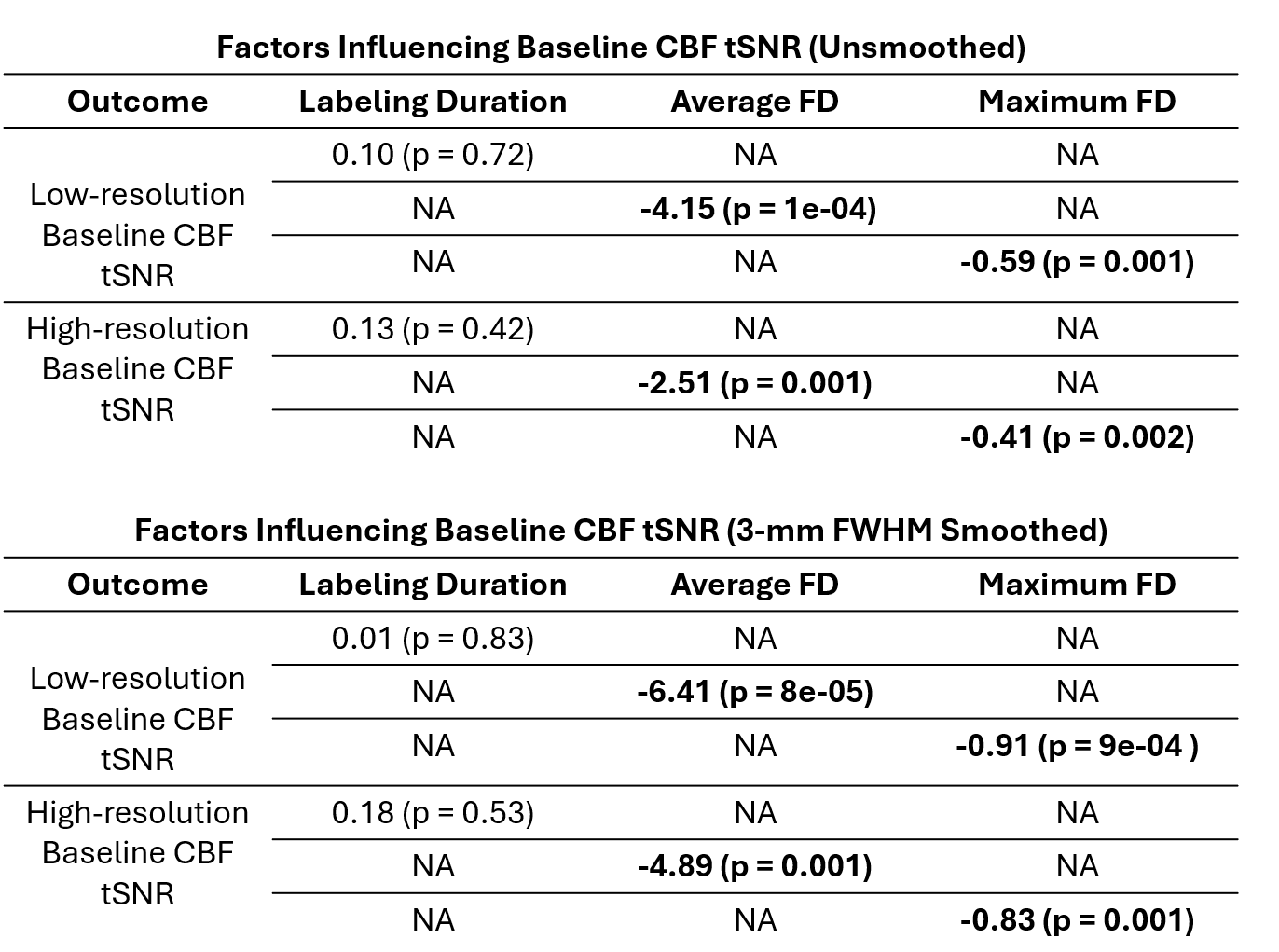


**Table S8**: Linear mixed effects models evaluating the factors influencing the temporal SNR (tSNR) of the low and high-resolution M2-PCASL baseline cerebral blood flow (CBF) time courses obtained from smoothing the M2-PCASL data with a 3-mm FWHM Gaussian filter. FD stands for framewise displacement. Bold font indicates statistical significance.
